# Secondary structure impacts patterns of selection in human lncRNAs

**DOI:** 10.1186/s12915-016-0283-0

**Published:** 2016-07-25

**Authors:** Cinta Pegueroles, Toni Gabaldón

**Affiliations:** 1 Bioinformatics and Genomics Programme, Centre for Genomic Regulation (CRG), The Barcelona Institute of Science and Technology, Dr. Aiguader 88, Barcelona, 08003 Spain; 2 Universitat Pompeu Fabra (UPF), Barcelona, 08003 Spain; 3 Institució Catalana de Recerca i Estudis Avançats (ICREA), Pg. Lluís Companys 23, Barcelona, 08010 Spain

**Keywords:** lncRNA, Purifying selection, Divergence, Polymorphism, Secondary structure

## Abstract

**Background:**

Metazoans transcribe many long non-coding RNAs (lncRNAs) that are poorly conserved and whose function remains unknown. This has raised the questions of what fraction of the predicted lncRNAs is actually functional, and whether selection can effectively constrain lncRNAs in species with small effective population sizes such as human populations.

**Results:**

Here we evaluate signatures of selection in human lncRNAs using inter-specific data and intra-specific comparisons from five major populations, as well as by assessing relationships between sequence variation and predictions of secondary structure. In all analyses we included a reference of functionally characterized lncRNAs. Altogether, our results show compelling evidence of recent purifying selection acting on both characterized and predicted lncRNAs. We found that RNA secondary structure constrains sequence variation in lncRNAs, so that polymorphisms are depleted in paired regions with low accessibility and tend to be neutral with respect to structural stability.

**Conclusions:**

Important implications of our results are that secondary structure plays a role in the functionality of lncRNAs, and that the set of predicted lncRNAs contains a large fraction of functional ones that may play key roles that remain to be discovered.

**Electronic supplementary material:**

The online version of this article (doi:10.1186/s12915-016-0283-0) contains supplementary material, which is available to authorized users.

## Background

Long non-coding RNAs (lncRNAs) are non-coding transcripts longer than 200 nt, which are often multiexonic and polyadenylated [1, 2]. Compared to protein coding genes, lncRNAs are transcribed at lower levels and tend to do so in a tissue-specific manner, which hampers their study and identification [3, 4]. So far, every search for lncRNAs in a metazoan genome has resulted in hundreds to thousands of predicted lncRNAs, with little overlap between studies. To date, most predicted lncRNAs remain without a known function. Nevertheless, there is a relatively small but steadily growing set of functionally characterized transcripts. LncRNAdb v2 [5], a reference database for functionally validated lncRNAs, lists 136 experimentally characterized human lncRNAs, and for some of them, the function and molecular mechanism are well characterized. For instance, *XIST* is involved in X chromosome inactivation for dosage compensation [6], *HOTAIR* interacts with the chromatin remodeling complex mediating epigenetic modifications of DNA [7], *H19* acts as a trans-regulator of imprinted genes [8], and *MALAT1* regulates alternative splicing and has been implicated in cancer [9, 10]. Other lncRNAs are only indirectly and loosely associated with a possible biological function. For instance, a recent study listed lncRNAs differentially expressed in normal and tumor samples but, for most of them, a direct implication in a biological process remains unclear [11].

The lack of a clear function for most lncRNAs, as well as their low levels of expression and sequence conservation, has led some authors to suggest that most lncRNAs may actually represent transcriptional “noise,” i.e., the result of non-specific transcription [12]. Validating this interpretation requires the assessment of selective constraints acting on human lncRNAs with a validated function. However, most previous studies have considered lncRNAs as a whole. Generally, these studies have found that, at the sequence level, lncRNAs are overall much less conserved than protein coding genes in all studied organisms [4, 13, 14]. Hallmarks of selection have been found in some organisms when comparing patterns of sequence variation in introns and exons of lncRNAs. For instance, a recent study detected selective pressures acting on lncRNAs of *Drosophila melanogaster* using both polymorphism and inter-specific conservation data [15]. For humans, by contrast, differences were weak or not significant (at the inter- and intra-specific levels, respectively) [15]. The authors suggested that due to the small human effective population size, selection is not strong enough to efficiently purge mutations on lncRNAs. Despite this, other studies have found that exons are more conserved than introns in human lncRNAs [16, 17]. Finally, some studies have noted that the lack of conservation is not constant across the entire sequence and that some lncRNAs contain highly conserved regions present across distant species [18–20]. A recent study showed that >85 % of lncRNAs had conserved splice sites that can be dated back to the divergence of placental mammals, despite a fast turnover of exons and introns [21]. It has been argued that these and other highly conserved elements may be related with the function of lncRNAs. Alternatively, however, these elements may play a role at the DNA level.

Secondary structure may be key for the function of lncRNAs, as supported by several independent analyses of some of the functionally characterized lncRNAs. For instance, in *MALAT1*, a highly conserved uracil-rich region contributes to RNA stability through the formation of a triple helix [22]. It has also been shown that the tumor suppressor function of the lncRNA *MEG3* can be attributed to two secondary fold motifs [23]. Some studies have found that specific folds in some lncRNAs, such as *SRA* [24] and *HOTAIR* [25], are conserved in distant species as a result of compensatory mutations. At the large scale, a genome-wide study based on 35 mammals detected that roughly 14 % of the *Homo sapiens* genome can fold into structures that are evolutionarily conserved and that most of them (88 %) fall in regions of low sequence conservation [26]. In addition, lncRNAs have been found to be stable as measured by their half-life, suggesting widespread functionality [27]. Finally, it has been observed that lncRNAs have a higher degree of secondary folding than predicted by chance, despite the fact that, surprisingly, lncRNAs seem to be less structured than mRNAs [28, 29]. Taken together, there is accumulating evidence that structure may play an important role in lncRNA functionality. However, it remains to be established on a genome-wide scale whether the patterns of secondary structure can effectively constrain sequence evolution in lncRNAs, particularly in species, such as human, with a low effective population size.

In conclusion, we still have a very poor understanding of how selective pressures may act on lncRNAs at the sequence and structural levels. Several key questions remain open that are central to the understanding of the evolution and function of lncRNAs. For instance, what are the signatures of selection in those lncRNAs which are known to have a function? What role does lncRNA secondary structure play in shaping sequence variation? And, finally, what fraction of annotated human lncRNAs is functional? To address these questions and gain further insights into what evolutionary pressures may be acting on lncRNAs, it is essential to combine evolutionary analyses at different levels. Firstly, inter- and intra-species level comparisons provide different degrees of resolution and are differentially affected by typical confounding factors such as the difficulties in aligning non-coding sequences. Secondly, given the lower sequence complexity of RNAs and their ability to maintain conserved structures despite high sequence variation, we consider it important to account for possible constraints at the structural level. Finally, given that a set of truly functional human lncRNAs exists, this can be exploited as a golden reference for establishing relationships between evolutionary constraints and functionality, thereby avoiding misleading comparisons with protein coding genes, whose functionality is achieved by decoding their sequence into proteins.

In this study, we focused on human intergenic lncRNAs to ensure that the observed sequence constraints were not influenced by overlapping protein coding genes. The studied lncRNAs were derived from GENCODE 19 [30] and were filtered with stringent criteria. We also used a control data set of truly functional and intergenic lncRNAs, consisting of 39 *H. sapiens* lncRNAs with an experimentally characterized biological function [31]. We analyzed patterns of sequence divergence, patterns of sequence polymorphism in different populations, and structural properties of these lncRNAs. In line with several previous studies, overall sequence conservation and single nucleotide polymorphism (SNP) density did not provide evidence of selection when comparing introns and exons. Finer and unprecedented analyses, however, revealed compelling evidence for purifying selection acting on functional lncRNAs in all human populations studied. Firstly, conserved elements were enriched in exons as compared to introns. Secondly, using population genetics parameters, we found that exons have an excess of low frequency polymorphisms as compared to introns. Finally, we found that SNPs are depleted in structured regions with low accessibility. This finding provides the first direct evidence of the impact of secondary structure in lncRNAs sequence variation. Importantly, these findings were also apparent for the bulk of predicted lncRNAs that remain uncharacterized, suggesting that the fraction of functional lncRNAs under selective constraint in this set is not negligible.

## Results and discussion

### Exons in lncRNAs are enriched in conserved elements but do not show overall higher conservation than introns

To provide a common background with previous studies using different sets of human lncRNAs, we first analyzed phastCons scores in exonic and intronic regions of lncRNAs and flanking protein coding genes, as well as in flanking intergenic regions. Since most human lncRNAs seem to be primate-specific [3, 4], we based our analysis on scores computed using an evolutionary model specific for primates (about 77 million years of evolution, according to TimeTree [32]). Strikingly, in the set of predicted lncRNAs (hereafter called the “broad set”) we observed that exons are significantly less conserved than introns and have similar levels of conservation as intergenic regions (Additional file 1: Figure S1). Thus, compared to a previous study using a 46-vertebrate model [15], we detected even fewer constraints, which may be due to the relatively poor quality of some primate genomes. This reinforces the idea that predicted human lncRNAs are in general very poorly conserved through evolution. However, this result may be due to the presence of noisily transcribed, non-functional transcripts in the broad set, and we expect larger constraints in functionally characterized lncRNAs. Indeed, a recent study using mouse (a species with a larger effective population size than human [33]) found that functional lncRNAs have levels of sequence constraint similar to those observed in protein coding genes [34]. However, according to the authors, some lncRNAs of their functional set overlapped with protein coding genes or were classified as “protein coding” in a previous study [4], which may have resulted in an overestimation of their conservation. Here we assessed conservation for the 39 human lncRNAs with an experimentally determined function (the “functional set”), which has been strictly filtered for any potential overlap with protein coding genes. We found that the functional and the broad sets show different distributions of phastCons score ratios in exons and introns (*P* = 0.004, Additional file 1: Figure S2). In contrast to the broad set, for functional lncRNAs we observed the expected pattern that exons are more conserved than introns, although these differences are not significant.

Since divergence estimates may be influenced by the presence of repeated elements, we calculated their abundance using the RepeatMasker software [35]. The percentage of sequences having repeats is quite similar when comparing the functional and broad sets, being slightly higher for the functional (71.79 %) than for the broad set (70.87 %). However, for those sequences having repeats, the percentage of sequences covered by interspersed repeats is higher for the broad (35.81 %) than for the functional (30.09 %) set. To evaluate whether these repeats are affecting our estimates, we also plotted phastCons scores for the best match (BM) subset of sequences having the same amount of mapped repeats (broad_BM: 351 sequences, Additional file 1: Figure S3, see Methods). In this later subset, differences between exons and introns were also significant, confirming previous results obtained using the entire broad set (Additional file 1: Figure S4). Thus, differences between the functional and the broad sets do not arise from different levels of repeated elements. Overall our results show that, contrary to what may be expected, conservation in lncRNAs proven to be functional is also very weak. This result implies that lack of inter-species conservation, as measured with this standard approach, cannot be taken as evidence of lack of functionality.

**Fig. 1:**
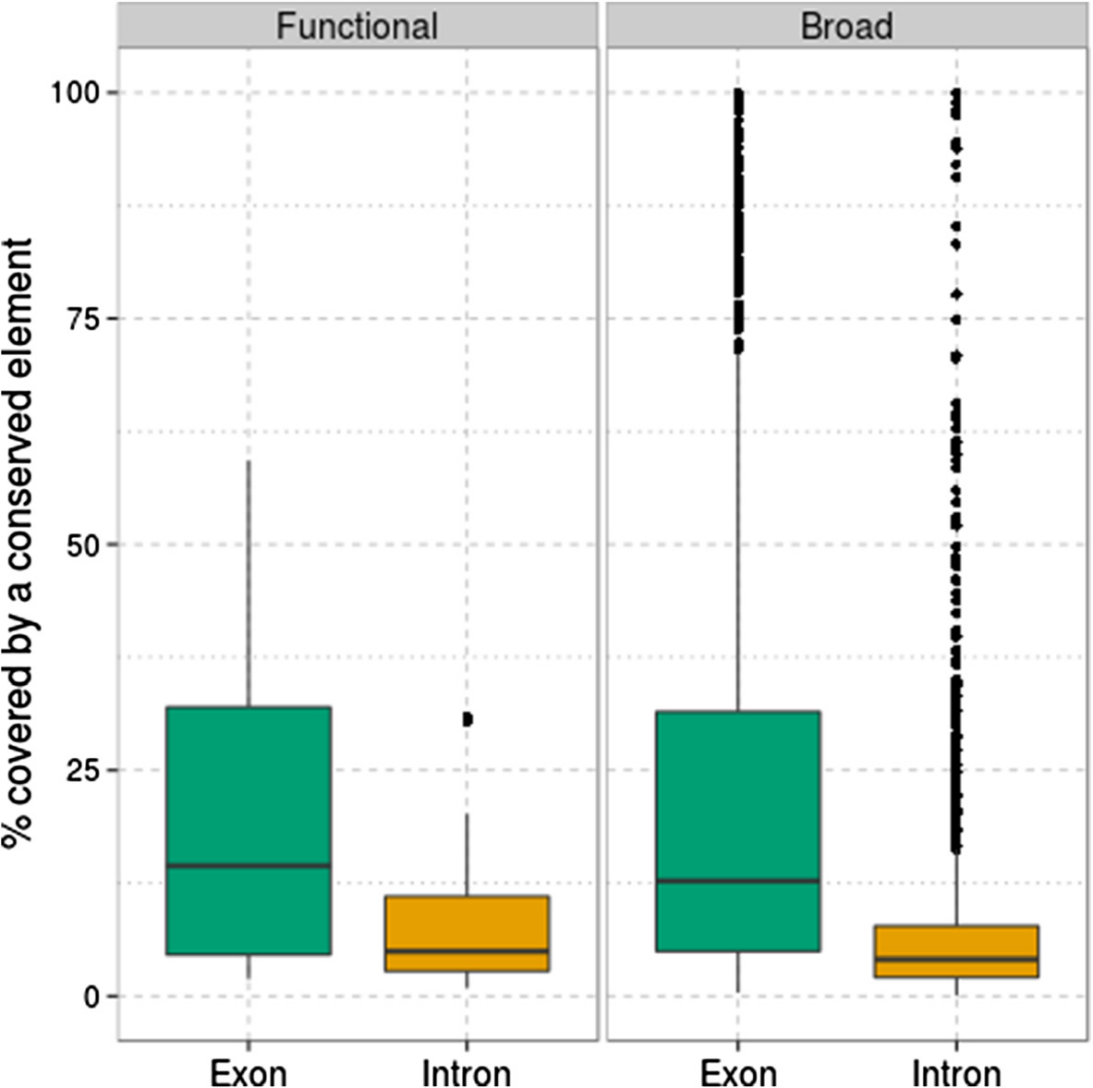
Boxplots showing the percentage of exonic and intronic sequences covered by conserved elements in the functional and broad human data sets. *Horizontal lines* inside *boxes* represent the median, *boxes* show the interquartile range (*IQR*, distance between first and third quartiles), *vertical lines* correspond to the *highest* and *lowest* value within 1.5*IQR, and *dots* represent outliers

As mentioned above, it has been suggested that short and highly conserved sequence elements may be involved in the function of lncRNAs, but it is as yet unclear whether these elements may play a role at the DNA level [1, 20, 36]. Other authors have proposed that conservation in lncRNAs is limited to splice-related motifs and that conservation in exon cores should be rare [29]. These models are compatible with observations of overall low sequence conservation. Indeed, if functionality of lncRNAs is conferred by short elements separated by largely unconstrained sequences, one could expect overall low conservation scores. In addition, if the observed conserved elements are indeed involved in lncRNA function, and not acting solely at the DNA level, one would expect them to specifically associate with exonic regions, thereby forming part of the mature lncRNA transcript. We compared the abundance of conserved elements, which are discrete regions having high conservation scores as predicted by phastCons, in both functional and broad human lncRNAs and using a multiple genome alignment of 100 vertebrates [37]. We observed that the percentage of lncRNAs covered by conserved elements is significantly higher in exons than in introns in both functional and broad data sets (*P* < 0.05, Fig. 1). These results support the idea that selective constraints may be limited to the maintenance of a few clusters of positions, which may be involved in lncRNA function by participating in structure or binding motifs present in the mature transcript.

### Human lncRNA exons show signatures of selection at the population level

Considering the low conservation of lncRNAs across species, it has been suggested that these molecules may have a high turnover and a short evolutionary lifespan [38]. If that is the case, selective constraints in functional lncRNAs may be stressed at the species or population level. We first focused on differences in SNP densities in exonic and intronic regions, which have been assessed before in the human African (AFR) population without finding significant differences [15]. We computed the SNP density in exons and introns in this and four other major human populations (Admixed American (AMR), European (EUR), East Asian (EAS), and South Asian (SAS)), which are roughly fourfold smaller than the AFR population in terms of effective size [39], and focused on differences between populations and between the broad and functional sets. The observed SNP density is fairly variable between populations, with the AFR and SAS populations having the highest and the lowest SNP density, respectively (Additional file 1: Figure S5), which is consistent with previous studies showing the highest genetic diversity in African populations [40, 41]. LncRNAs and intergenic regions have higher SNP densities, as compared to protein coding genes, and differences between them are generally not significant (Additional file 1: Figure S5). The distributions of SNP densities in the functional and broad sets are not significantly different (Additional file 1: Figure S6). In the two sets, we observed that exons tend to accumulate fewer SNPs than introns, but differences were only significant for some populations in the broad set (AMR and SAS, Additional file 1: Figure S7). Thus, our results are generally in line with those of a previous study restricted to the AFR population [15]. However, our results reveal that lncRNAs with a known function display similarly low differences in SNP densities between exons and introns; therefore, this feature cannot be used as evidence for a lack of functionality.

**Fig. 2:**
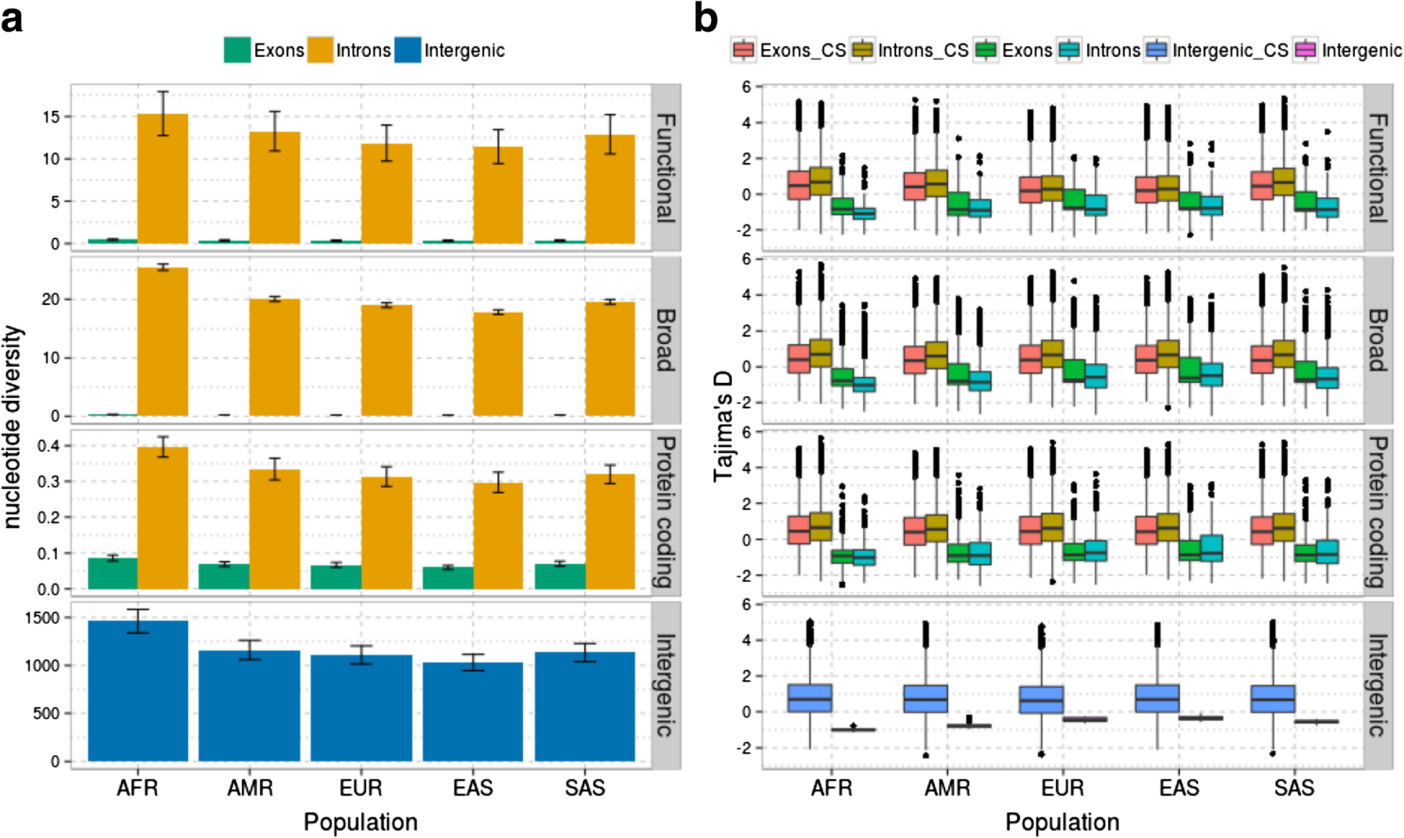
**a** Mean nucleotide variability (π) for exonic and intronic regions of the functional and broad lncRNA sets in human and nearby protein coding genes and intergenic regions. Error bars represent the standard error of the mean. **b** Tajima's *D* values for exonic and intronic regions for the functional and broad lncRNA in human and nearby protein coding genes and intergenic regions. Tajima's *D* values were computed for observed values (*green*, *blue*, and *pink*) and coalescence simulations (*CS*, in *red*, *yellow*, and *light blue*). *Horizontal lines inside boxes* represent the medians, *boxes* show the interquartile range (*IQR*, distance between first and third quartiles), *vertical lines* correspond to the highest and lowest values within 1.5*IQR, and *dots* represent outliers

To gain a deeper insight into the selective pressures acting on human lncRNAs, we performed a more thorough analysis by estimating several population genetics parameters, including nucleotide diversity (π), derived allele frequency (DAF), and Tajima's *D*. Nucleotide diversity (π) is defined as the average number of pairwise nucleotide differences per site [42]. Figure 2a shows the nucleotide diversity of the two sets of human lncRNAs, as well as that of surrounding protein coding genes and intergenic regions. We made three major observations. First, nucleotide diversity levels are different between the four categories: intergenic regions and protein coding exons show the highest and lowest levels of genetic diversity, respectively, and the broad set of lncRNAs has higher values than the functional set. Second, levels of nucleotide diversity vary among populations, and they can be ordered from highest to lowest levels (AFR, AMR, SAS, EUR, and EAS, in this order), and the order is the same in the four categories studied. Of note, the lowest levels of SNP density in the SAS population are not related with the lowest π levels, since SAS has higher π levels than EUR and EAS populations. Third, we observed, for the first time in human populations, that nucleotide diversity is significantly smaller in exons than in introns in both functional and broad lncRNA sets. We also evaluated whether the differential levels of repeats in the functional and broad sets are biasing our results, computing π for a subset of broad lncRNAs having the same amount of mapped repeats (broad_BM). The levels of π are similar to those for the whole set and are significantly lower in exons compared to introns, indicating that the differential composition of repeats in the sets is not biasing our results (Additional file 1: Figure S8a). Overall, in human lncRNAs, SNP density and nucleotide diversity seem to be subjected to different degrees of constraint, and only nucleotide diversity has robust detectable differences between exonic and intronic sequences.

To further evaluate whether the observed genetic diversity patterns deviate from neutrality expectations, we performed Tajima's *D* tests [43]. Tajima's *D* is calculated as the difference between two measures of genetic diversity: the mean number of pairwise differences and the number of segregating sites, each scaled so that they are expected to be equal in a neutrally evolving population of constant size. Tajima's *D* was calculated for each data set (lncRNA and surrounding protein coding genes and intergenic regions) and for coalescent simulations that were computed using the observed population mutation rate value (theta) for each region and a basic model (constant population size, no recombination, panmixis, and an infinite-sites model) with the ms program [44]. Tajima's *D* values were negative in the four data sets (the two sets of human lncRNAs and surrounding protein coding genes and intergenic regions) and in all five populations studied (Fig. 2b). Tajima's *D* values in the broad_BM subset were similar to those for the whole broad set, indicating that the differential composition of repeats in the sets is not biasing our estimates (Additional file 1: Figure S8b). The observed Tajima's *D* values are different from those obtained in the coalescence simulations, supporting the hypothesis that observed values deviate from neutral expectations due to an excess of polymorphism at low frequency. The bias towards low frequency variants in lncRNAs was confirmed in both exonic and intronic regions when evaluating the DAF (Additional file 1: Figure S9). Deviations from neutral expectations may be interpreted as the consequence of a recent population bottleneck and/or purifying selection. Human populations are known to have undergone a recent expansion [40, 45], which may contribute to the negative Tajima's *D* values detected in all regions studied, including intronic and surrounding intergenic regions. However, we also detected that π is not uniformly distributed in exonic and intronic regions and also not between lncRNAs, protein coding genes, and intergenic regions. Thus, selective constraints contribute to the observed deviations from neutral expectations. Taken together, our results suggest that purifying selection may be acting on human lncRNAs to prevent the accumulation of deleterious mutations, in both the functional and broad sets.

### Secondary structure constrains sequence variation in lncRNAs

It has been proposed that some lncRNAs may function through the adoption of specific secondary structure folds [46]. In a previous study, the presence of a high number of correlated positions on multiple alignments was interpreted as evidence of evolutionary conservation of RNA secondary structures [17]. We evaluated the secondary structure of human lncRNAs, rRNA, mRNA, and intergenic regions using accessibility scores calculated with two independent methods, which indicate the probability that each site belongs to an unpaired region according to an ensemble of computationally predicted secondary structures (see Methods). rRNAs should be considered as a positive control, since their functionality is known to depend on their secondary structure. By contrast, intergenic regions should be considered as a negative control, since their function (if any) is not expected to be driven by their RNA secondary structure. Although the function of mRNAs depends primarily on the encoded protein, protein coding transcript sequences have been shown to be constrained at the structural level [28]. Regardless of the method used to calculate accessibilities, all data sets had similar distributions of residue accessibility, in which non-accessible residues likely to be paired or close to paired residues constitute the largest fraction (Additional file 1: Figure S10).

**Fig. 3:**
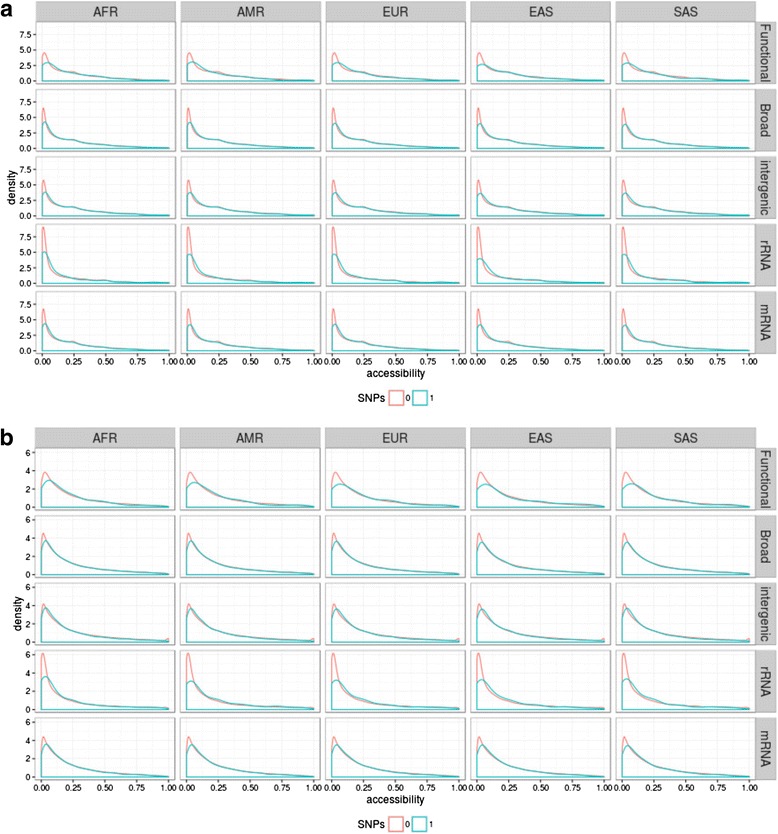
Density plots showing the accessibility distribution for positions containing or not containing an SNP in the five major populations: African (*AFR*), Ad Mixed American (*AMR*), European (*EUR*), East Asian (*EAS*), and South Asian (*SAS*). Accessibility was computed using the Sfold (**a**) and the RNAfold (**b**) programs

**Fig. 4:**
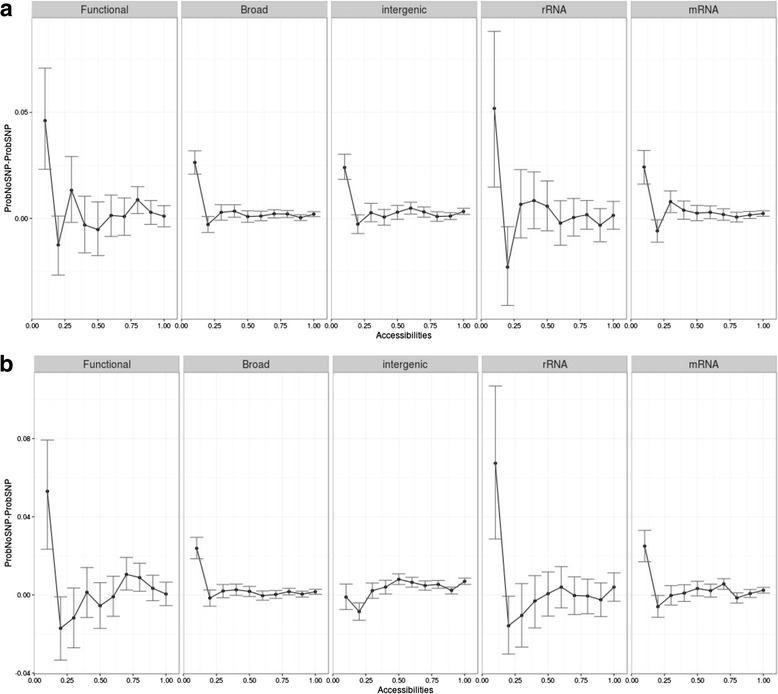
Difference between accessibility distributions of positions with or without SNP within a given range of accessibilities (0–0.1, 0.1–0.2, 0.2–0.3, 0.3–0.4, 0.4–0.5, 0.5–0.6, 0.6–0.7, 0.7–0.8, 0.8–0.9, 0.9–1). Probabilities within ranges were calculated using the integrate.xy function on a density distribution (see Methods). *Vertical lines* represent the confidence intervals estimated using a bootstrapping after 1000 replicates. Accessibility was computed using the Sfold (**a**) and the RNAfold (**b**) programs using SNPs from the African (*AFR*) population (see Additional file 1: Figure S12 for other populations)

Firstly, we evaluated whether conserved positions (i.e., those positions included in a phastCons conserved element) and non-conserved positions have different accessibilities. The distributions of accessibilities in conserved and non-conserved positions are significantly different in the functional set (*P* < 0.001 for both Sfold and RNAfold estimates after a Wilcoxon test) but not in the broad set. However, when computing the median accessibilities for conserved and non-conserved positions for each lncRNA, differences remain significant only for the Sfold method (*P* = 0.03, Additional file 1: Figure S11). These results suggest that conserved elements may be enriched in secondary structure folds, which in turn may be related to their function. Secondly, to evaluate whether the secondary structure influences the location of SNPs, we calculated the prevalence of polymorphic sites at positions with different accessibilities. We observed that positions of low accessibility showed lower probabilities of having SNPs (Fig. 3). Importantly, in the rRNA, functional, broad, and mRNA data sets, the differences between the distributions of positions with SNP or without them were significant and larger in the range of positions with very low accessibilities (between 0 and 0.1) than in the rest of the accessibility ranges, independent of the method used to calculate accessibilities (Fig. 4, Additional file 1: Figure S12). These low accessibility positions are likely to be paired or close to paired residues and constitute the largest fraction (Additional file 1: Figure S10). Note that accessibilities independently computed using the two different softwares behave in the same way for all sets, the only exception being the intergenic regions. According to the RNAfold program intergenic regions do not show a tendency to prevent the accumulation of SNPs in low accessibilities, while according to the Sfold program the behavior of the intergenic regions is similar to that of the broad and mRNA regions. These results suggest that the secondary structures predicted in the intergenic regions should be considered with caution. Importantly, both programs show that the differences between this particular range of accessibilities and others are particularly stressed in both the rRNA and the functional sets. This indicates that, overall, SNPs are prevented from accumulating in positions of low accessibility, that is, positions in paired regions that participate in the formation of secondary structure folds, and therefore may be key in achieving their function.

Some of the lncRNAs may be partially annotated, and this may affect the predictions of the secondary structure. Thus, we selected a subset of putative full-length transcripts by keeping those that had the same length in GENCODE 19 and 24, which is the latest release. The subsets resulted in 35 out of 38 for the functional lncRNA set and 3394 out of 3483 for the broad lncRNA set. In both cases we detected the same trend as obtained when using the whole data set, with SNPs prevented from accumulating in regions with low accessibility (Additional file 1: Figure S13). Thus, the presence of partially annotated genes does not seem to affect our estimates of accessibility.

To evaluate whether our results are biased due to the nucleotide composition of the sequence context, we compared GC content (% GC) with the mean number of SNPs and the accessibility scores (Additional file 1: Figure S14). The three parameters (% GC, mean SNPs, and mean accessibilities) were calculated for non-overlapping windows of five nucleotides. As expected, we observed a negative correlation between % GC and accessibility, confirming previous results [47, 48]. Importantly, the mean number of SNPs remains similar for different values of % GC, indicating that the observed depletion of SNPs in low accessibility sites does not depend on GC content.

Previous studies showed that purifying selection is maintaining a splice-related motif, i.e., an exonic splicing enhancer (ESE), near exon boundaries to ensure an efficient splicing of multiexonic lncRNA [29, 49]. Schüler et al. [29] concluded that purifying selection acts to maintain ESE motifs but not necessarily RNA folding, since they failed to find a correlation between evolutionary rate and secondary structure stability. In our study we detected that SNP density is lower in ESE motifs than in non-ESE regions, and differences were significant for the broad set in the five populations studied (Additional file 2: Table S3), providing additional support to the idea that constraints are larger in ESE than in non-ESE regions. We wanted to test whether the observed relationship between accessibility and SNP density is due to the presence of ESE motifs, which may point to splicing as the main factor driving the observed relationships between conservation and structure. To this end we classified the positions of lncRNAs according to the presence or not of ESE motifs, and we compared the accessibility distributions for positions not having and having SNPs (Additional file 1: Figure S15). Overall the behavior of the sites with or without annotated ESEs is similar for both the Sfold and RNAfold programs, although in the broad set differences are higher for the ESE positions in all populations studied. Thus, the reduction of SNPs in positions of low accessibility cannot be solely explained by the presence of ESE elements. Altogether, our results suggest that secondary structure constrains ancient and recent sequence variation in lncRNAs, and that this is largely independent of the presence of known motifs involved in splicing.

**Fig. 5:**
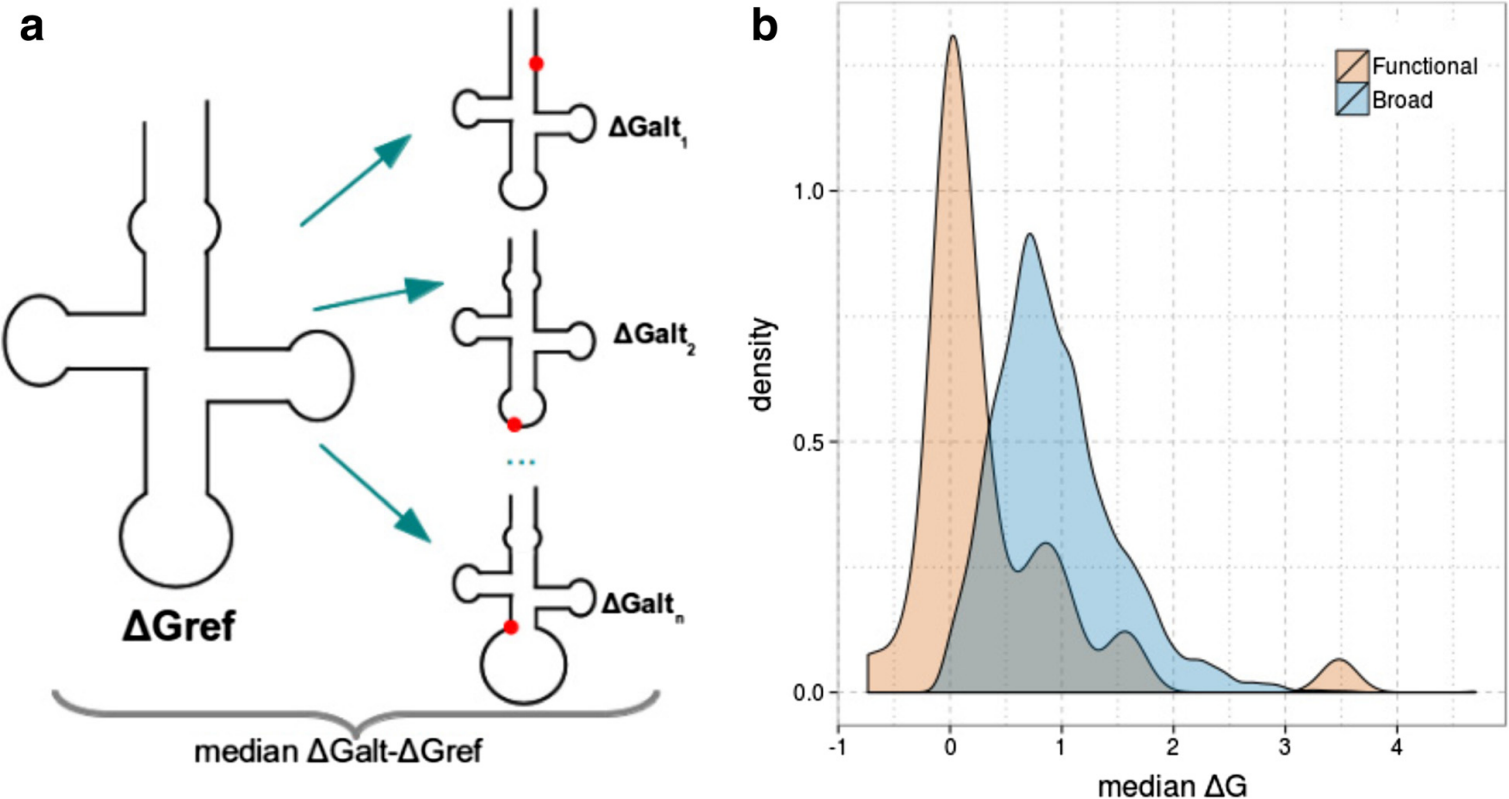
**a** Diagram showing how median Δ*G* was calculated for each lncRNA, which is based on the Δ*G* of the native structure and the structure with SNPs (*red dots*). **b** Density plot showing the median values of Δ*G* for the functional (*red*) and broad (*blue*) human sets

Finally, an alternative way to measure whether SNPs that impair folding are purged by natural selection is to estimate the impact of the variation on the energetic stability of the fold. We did so by comparing the minimal Gibbs free energy (Δ*G*) of the reference structure and the structure of the lncRNA having a certain SNP, as reported in the lncRNASNP database [50] (Fig. 5a, b). The density plots are significantly different in the two sets (*P* = 1.41e-11). Notably, in the functional data set, median values of the change in minimal energy are narrowly centered around zero, suggesting that SNPs located in functional lncRNAs do not generally affect the stability of the secondary structure. Conversely, in the broad set, energy changes are shifted to positive values, suggesting that SNPs accumulated in these lncRNAs may result in less stable secondary structures. To our best knowledge, this is the first study that provides compelling evidence for an impact of secondary structure on lncRNA sequence variation.

## Conclusions

We have found evidence of selection acting on lncRNAs at both sequence and structural levels. When evaluating divergence data, which include ancient events, we observed that exons are observably but not significantly more conserved in exons compared to introns in the functional set. Interestingly, in both functional and broad sets, we observed a significant enrichment of conserved elements in exonic regions which may be related with lncRNA functionality. When evaluating more recent events using sequence polymorphisms, we found evidence that purifying selection prevents increases in the frequency of slightly deleterious mutations, especially in exonic regions, in both functional and broad sets. Furthermore, in lncRNAs with an experimentally characterized function we found that structural regions with low accessibility are more likely to be conserved. In addition, we observed that in lncRNAs, mRNAs, and rRNAs, segregating sites are prevented from accumulating in low accessibility, paired regions, and SNPs in functional lncRNAs had little impact on the stability of the secondary structure. Importantly, these results are independent of the GC content, the presence of ESE motifs, and the presence of partial sequences. Taken together, these results suggest that, overall, lncRNA structure introduces constraints on the evolution of its sequence.

We have observed that functional and broad human lncRNAs have different evolutionary constraints, although in both sets nucleotide diversity is driven by recent purifying selection. The functional set is generally more conserved, especially in exons, and secondary structure may be maintained through constraints on SNP location. In the broad set, selective constraints are generally weaker at both the sequence and secondary structure levels. Despite these overall differences, it is difficult to predict the functionality of an individual lncRNA based on the observed sequence or structural constraints, since there is a great variation in each of these single values. This indicates that the set of functionally characterized human lncRNAs is a heterogeneous group, with respect to their evolutionary signatures. Heterogeneity in the functional set may be a consequence of the different functions in which they are involved. Note that, for most parameters studied, the functional and broad sets have overlapping distributions, suggesting that numerous lncRNAs of the broad set may be functional.

In summary, our study provides new evidence that lncRNAs are subjected to purifying selection in human populations, and therefore numerous predicted lncRNAs are potentially functional. In addition we found first evidence that secondary structure of lncRNAs shapes recent sequence variation. In general, conservation is low in lncRNAs exons but remains detectable in short, discrete regions, which have a higher tendency to participate in structural folds. Altogether our results support a model in which the functionality of lncRNAs can be maintained despite large sequence divergence, probably by maintaining the presence of short elements, likely involved in folding and other forms of functionality, which are surrounded by loosely constrained regions that may act as spacers. Future experimental analyses are needed to determine whether those short conserved regions are actually functional in the mature lncRNA.

## Methods

### Selection of intergenic lncRNA and flanking intergenic regions and protein coding genes

We considered 12,101 lncRNA transcripts, annotated in Ensembl r75, derived from GENCODE 19, and we filtered them by applying a strict pipeline. In this pipeline, transcripts were discarded if they were (1) shorter than 199 nt, (2) repeated (i.e., transcripts having a different identifier but identical sequence), (3) overlapping any protein coding genes annotated in Ensembl, (4) exhibiting coding potential according to the CPC software [51], or (5) monoexonic. After applying our pipeline, we kept 5245 transcripts corresponding to 3741 genes, hereafter called the broad set. For each lncRNA in this set, we retrieved the sequences from regions falling within 5 kb upstream and downstream of the lncRNA gene. First, we obtained a bed file including all annotated genes in Ensembl r75 and our lncRNA list. Then, we obtained a bed file including all unannotated regions of each genome using the substractBed tool in BEDTools v2 [52], hereafter defined as intergenic regions. Similarly, we selected exons and introns belonging to protein coding genes located within 5 kb upstream and downstream of each lncRNA, referred to as the mRNA data set. Additionally, we considered a second data set of functional lncRNAs annotated in lncRNAdb v2 [31]. We removed lncRNAs overlapping with any of the protein coding genes annotated in Ensembl r75 and those that were monoexonic to obtain a final list of 39 functionally validated lncRNA genes, which are referred to as the “functional set” throughout the text.

### Sequence conservation of lncRNA across species

The phastCons scores [37] were retrieved from the UCSC database [53]. We then calculated average phastCons scores for each exonic and intronic region of each transcript, using the bigWigAverageOverBed tool and computed the average phastCons score per transcript. The phastCons scores were computed using genomic alignments of 46 vertebrate species and a tree model for primates (including human, chimp, gorilla, orangutan, rhesus, baboon, marmoset, tarsier, mouse lemur, and bushbaby). We discarded 216 out of 5245 transcripts after filtering by requiring the presence of a minimum of two species in the genomic alignment. The remaining 5029 lncRNA transcripts (3597 genes) have a median 53 % identity. Sixteen of them were further discarded because they were already included in the functional set. We selected the longest transcript of each lncRNA to perform further analyses. Transcript IDs and genomic locations of the longest transcript of the selected lncRNAs for each species are shown in Additional file 2: Tables S1 and S2. Finally, we calculated average phastCons scores for intergenic regions and protein coding genes located within 5 kb of the selected lncRNA (see above). We also retrieved a list of phastCons conserved elements from UCSC Table Browser [54] that were annotated using a multiple genome alignment of 100 vertebrates [55].

### Sequence polymorphism

The polymorphism data were downloaded from phase 3 data from the 1000 Genomes Project [56]. We extracted data from five super-populations: African (AFR; 42,486,664 SNPs), Admixed American (AMR; 26,968,342 SNPs), European (EUR; 23,123,795 SNPs), East Asian (EAS; 22,899,456 SNPs), and South Asian (SAS; 25,745,962 SNPs). For each species and population, we mapped SNPs to the longest isoforms of lncRNAs and flanking protein coding genes, and to the flanking intergenic regions. We computed the derived allele frequency (DAF) [57], the nucleotide diversity (π), and Tajima's *D* for exonic and intronic regions of the longest transcript of each lncRNA using PopGenome [58]. Because of technical issues, chromosome Y and chromosome X of males were discarded in the PopGenome analyses. Finally we computed 1000 coalescent simulations for each chromosome using the observed population mutation rate value (theta) and a basic model (constant population size, no recombination, panmixis, and an infinite-sites model) with the ms program [59]. Because of the high number of SNPs mapped in intergenic regions, the number of coalescent simulations in this latter set was limited to 500 per chromosome.

### Secondary structure

We calculated the residue accessibility levels (probability of a residue and their neighbors to be unpaired in the folded RNA) for the lncRNA, intergenic regions, and mRNA data sets, as well as for a set of 566 human rRNAs. In the intergenic data set we discarded the regions located less than 1 kb from the lncRNA to minimize the presence of possible UTR regions. For these, sequence fragments were created from lncRNA transcripts using overlapping windows of 80 nt with an increment of 20 nt over the entire transcript. For each fragment Sfold [60] was used to sample 1000 secondary structures and compute residue accessibilities. Residue accessibility was calculated for each position (*i*) by averaging the values obtained for all fragments as reported by Sfold and using a window of four nucleotides: the accessibility is the probability that nucleotides *i*, *i* + 1, *i* + 2, and *i* + 3 are all unpaired (*W* = 4). Similarly, we calculated accessibility using the program RNAfold [61] using windows of four nucleotides. We also calculated the percentage of GC, the mean number of SNPs, and mean accessibility in non-overlapping windows of five nucleotides. We used the density function in the stats package for R to calculate the probability distributions for positions having and not having SNPs, and the area under the curve between two given accessibility values was calculated using the integrate.xy function from the sfsmisc package. Confidence intervals were estimated using a bootstrapping strategy, implemented using the boot package from R. Furthermore, we classified positions as being covered or not by an ESE motif using the same experimentally confirmed set as Schüler et al. [29], and we did the same analyses as with the whole data sets. We also retrieved the minimal Gibbs free energy (∆*G*) as calculated in lncRNASNP for *H. sapiens* lncRNA [50]. For each lncRNA, the database provides the secondary structure and the minimum free energy (∆*G*) of the folded reference transcript sequence and that obtained after replacing each SNP annotated in dbSNP. Using these data, we calculated the median ∆*G* for functional and non-functional *H. sapiens* lncRNAs.

### Sequence repeats

The presence of repeated elements was evaluated using RepeatMasker software using default parameters. For each lncRNA, we calculated the percentage of sequences covered by six major types of repeats: SINEs, LINEs, LTRs, DNA elements, simple repeats, and low complexity. To optimally select a subset of lncRNA from the broad set having the same abundance of repeated elements, we used the nbpMatching package for R. Briefly, for all lncRNA from the functional set, we found the best matches in the broad set according to their composition in repeats, and we removed this set of lncRNA from the analysis. Using this procedure selected the 10 % of sets having the best matches, consisting in 351 sequences (broad_BM, Additional file 1: Figure S3).

### Statistical tests and plots

All statistical tests and plots were performed using the R statistical software package [62]. The Wilcoxon test was computed with default parameters and used in pairwise comparisons between exonic and intronic distributions. We corrected for multiple testing using the Benjamini and Hochberg method [63]. Plots were produced using the ggplot2 package in R [64].

## Abbreviations

AFR, African; AMR, Admixed American; DAF, derived allele frequency; EAS, East Asian; EUR, European; lncRNA, long non-coding RNA; SAS, South Asian

## Additional files

Additional file 1: Figures S1–S15.
** Figure S1.** Boxplots showing mean phastCons scores for exons and introns of lncRNAs, protein coding genes, and intergenic regions located within 1 and 5 kb from any of the lncRNAs. **Figure S2.** Density plots showing the distribution of differences in the mean phastCons scores of exons and introns computed for each lncRNA. **Figure S3.** Proportion of repeats found in the functional, broad, and best matches subset (broad_BM). **Figure S4.** Boxplots showing mean phastCons scores for exons and introns of lncRNAs for the functional, broad human and broad_BM data sets. **Figure S5.** Median SNP density and 95 % confidence interval for exonic and intronic regions of lncRNA, nearby protein coding genes (1 and 5 kb), and surrounding intergenic regions (1 and 5 kb). **Figure S6.** Density plots showing differences in SNP density of exons minus introns computed for each lncRNA. **Figure S7.** Boxplots showing SNP density in exons and introns for the five major human populations in the functional and the broad sets. **Figure S8.** Mean nucleotide variability for exonic and intronic regions of broad and broad_BM sets. **Figure S9.** Derived allele frequency for exonic and intronic regions of the functional and broad human data sets in the five major populations. **Figure S10.** Comparison of the accessibility distribution in the functional, broad, intergenic, rRNA, and mRNA sets. **Figure S11.** Median accessibility for conserved and non-conserved positions. **Figure S12.** Differences between accessibility distributions of positions with or without SNPs within a given range of accessibilities. **Figure S13.** Differences between accessibility distributions of positions with or without SNPs for the whole functional and broad sets and for those lncRNAs likely to be fully annotated, within a given range of accessibilities. **Figure S14.** Boxplots showing the correlation between the mean accessibility and the mean % GC, and the mean number of SNPs and the mean % GC, calculated in non-overlapped windows of five nucleotides for both the functional and the broad sets in the five major populations. **Figure S15.** Differences between accessibility distributions of positions with or without SNPs for the functional and the broad sets. (PDF 8535 kb) Additional file 2: Tables S1–S3.
** Table S1.** Chromosome, transcript start coordinates, transcript end coordinates, and transcript ID for each lncRNA of the functional set. **Table S2.** Chromosome, transcript start coordinates, transcript end coordinates, and transcript ID for each lncRNA of the broad set. **Table S3.** Mean number of SNPs in regions classified according to the presence or not of ESE motifs for the functional and broad human sets in the five major populations. (XLS 295 kb)
