# Supplementary material for: Secondary structure impacts patterns of selection in human lncRNAs
Source: BMC Biol. 2016 Jul 25;14:60. doi: 10.1186/s12915-016-0283-0 (PMC4960838; doi:10.1186/s12915-016-0283-0)
Supplement: Additional file 1: Figures S1–S15. — Figure S1. Boxplots showing mean phastCons scores for exons and introns of lncRNAs, protein coding genes, and intergenic regions located within 1 and 5 kb from any of the lncRNAs. Figure S2. Density plots showing the distribution of differences in the mean phastCons scores of exons and introns computed for each lncRNA. Figure S3. Proportion of repeats found in the functional, broad, and best matches subset (broad_BM). Figure S4. Boxplots showing mean phastCons scores for exons and introns of lncRNAs for the functional, broad human and broad_BM data sets. Figure S5. Median SNP density and 95 % confidence interval for exonic and intronic regions of lncRNA, nearby protein coding genes (1 and 5 kb), and surrounding intergenic regions (1 and 5 kb). Figure S6. Density plots showing differences in SNP density of exons minus introns computed for each lncRNA. Figure S7. Boxplots showing SNP density in exons and introns for the five major human populations in the functional and the broad sets. Figure S8. Mean nucleotide variability for exonic and intronic regions of broad and broad_BM sets. Figure S9. Derived allele frequency for exonic and intronic regions of the functional and broad human data sets in the five major populations. Figure S10. Comparison of the accessibility distribution in the functional, broad, intergenic, rRNA, and mRNA sets. Figure S11. Median accessibility for conserved and non-conserved positions. Figure S12. Differences between accessibility distributions of positions with or without SNPs within a given range of accessibilities. Figure S13. Differences between accessibility distributions of positions with or without SNPs for the whole functional and broad sets and for those lncRNAs likely to be fully annotated, within a given range of accessibilities. Figure S14. Boxplots showing the correlation between the mean accessibility and the mean % GC, and the mean number of SNPs and the mean % GC, calculated in non-overlapped windows of five nucleotides for bo [file 12915_2016_283_MOESM1_ESM.pdf]

Figure S1: Boxplots showing mean phastCons scores for exons and introns of lincRNAs, protein coding genes and intergenic regions located within 1 and 5kb from any of the lincRNA (protCod1kb/P1kb, protCod5kb/P5kb, i1kb and i5kb, respectively) for the functional and broad human data sets. Horizontal lines inside the boxes represent the median, boxes show the interquartile range (IQR, distance between first and third quartiles), vertical lines correspond to the highest and lowest value within 1.5*IQR and dots represent outliers. Heatmaps show the adjusted *P* value after Wilcoxon test and red stars indicate statistically significant differences in the comparison.

Figure S2: Density plots showing the distribution of differences in the mean phastCons scores of exons and introns computed for each lincRNA. The two density plots correspond to the functional (red) and broad (blue) human lincRNA sets. Dashed-vertical line designates mean Phastcons exons = introns (i.e. exons - introns = 0). Values at the right of the vertical bar are cases in which exons > introns (i.e. exons - introns >0); values at the left of the vertical bar are cases in which exons < introns (i.e. exons - introns <0).

Figure S3: Proportion of repeats found in the functional, broad and best matches subset (Broad_BM). Broad_BM is a subset of 351 sequences having the same levels of repeated elements than the functional set (see Materials and Methods). The proportion of repeats was calculated as the mean percentage of sequence covered by a certain type of repeated element divided by the mean percentage of sequence covered by repeats, for each data set.

Figure S4: Boxplots showing mean phastCons scores for exons and introns of lincRNAs for the functional, broad human and Broad_BM data sets. Horizontal lines inside the boxes represent the median, boxes show the interquartile range (IQR, distance between first and third quartiles), vertical lines correspond to the highest and lowest value within 1.5*IQR and dots represent outliers. Broad_BM is a subset of 351 sequences having the same levels of repeated elements than the functional set (see Materials and Methods).

Figure S5: Above: median SNP density (i.e. number of SNPs / length in nucleotides) and 95% confidence interval for exonic and intronic regions of lincRNA, nearby protein coding genes (1 and 5 kb) and surrounding intergenic regions (1 and 5 kb). Below: p-values after Wilcox test. For the human data set we plotted (a) African, (b) Ad Mixed American, (c) European, (d) East Asian and (e) South Asian populations.

Figure S6: Density plots showing differences in SNP density (i.e. number of SNPs/ length) of exons minus introns computed for each lincRNA. in the functional (red) and broad (blue) sets. Plots were computed for the five major human populations: African (AFR), Ad Mixed American (AMR), European (EUR), East Asian (EAS) and South Asian populations (SAS). Table shows *P* value after comparing the functional and the broad sets within each population using Wilcoxon test. Vertical line designates mean SNP density exons = introns (i.e. exons - introns = 0). Values at the right of the vertical bar are cases in which SNP density exons > introns (i.e. exons -introns >0); values at the left of the vertical bar are cases in which SNP density exons < introns (i.e. exons -introns <0).

Figure S7: Boxplots showing SNP density (i.e. number of SNPs/ length) in exons (green) and introns (yellow) for the five major human populations (African (AFR), Ad Mixed American (AMR), European (EUR), East Asian (EAS) and South Asian (SAS)) in the functional (a) and the broad (b) sets. Horizontal line inside boxes represents the median, boxes show the interquartile range (IQR, distance between first and third quartiles), vertical lines correspond to the highest and lowest value within 1.5*IQR and dots represent outliers. Table shows median values within each category and *P* value after applying a Wilcoxon test.

Figure S8: (a) Mean nucleotide variability (π) for exonic and intronic regions of broad and broad_BM sets. Error bars represent the standard error of the mean. (b) Tajima's D values for exonic and intronic regions for broad and broad_BM sets. Horizontal lines inside boxes represent the medians, boxes show the interquartile range (IQR, distance between first and third quartiles), vertical lines correspond to the highest and lowest value within 1.5*IQR and dots represent outliers. Broad_BM is a subset of 351 sequences having the same levels of repeated elements than the functional set (see Materials and Methods).

Figure S9: Derived allele frequency (DAF) for exonic (green) and intronic (yellow) regions of the functional and broad human data sets in the five major populations: African (AFR), Ad Mixed American (AMR), European (EUR), East Asian (EAS) and South Asian populations (SAS).

Figure S10: Comparison of the accessibility distribution in the functional, broad, intergenic, rRNA and mRNA sets for values obtained using the Sfold program (a) and the RNAfold program (b).

Figure S11: Median accessibility for conserved (red) and non-conserved (blue) positions calculated using the Sfold and the RNAfold programs. Horizontal line inside boxes represents the median, boxes show the interquartile range (IQR, distance between first and third quartiles), vertical lines

correspond to the highest and lowest value within 1.5*IQR and dots represent outliers.

Figure S12: Differences between accessibility distributions of positions with or without SNPs within a given range of accessibilities (0-0.1, 0.1-0.2, 0.2-0.3, 0.3-0.4, 0.4-0.5, 0.5-0.6, 0.6-0.7, 0.7-0.8, 0.8-0.9, 0.9-1). Probabilities within ranges were calculated using the integrate.xy function on a density distribution (see material and methods). Vertical lines represent the confidence intervals estimated using a bootstrapping after 1000 replicates. Accessibility was computed using the Sfold program for the AMR (b), EUR (c), EAS (d) and SAS (d) populations and the RNAfold program for the AMR (e), EUR (f), EAS (g) and SAS (h) populations

Figure S13: Differences between accessibility distributions of positions with or without SNPs for the whole functional and broad sets (in blue) and for those lncRNA likely to be fully annotated (in red, see results section), within a given range of accessibilities (0-0.1, 0.1-0.2, 0.2-0.3, 0.3-0.4, 0.4-0.5, 0.5-0.6, 0.6-0.7, 0.7-0.8, 0.8-0.9, 0.9-1). Probabilities within ranges were calculated using the integrate.xy function on a density distribution (see material and methods). Vertical lines represent the confidence intervals estimated using a bootstrapping after 1000 replicates. Accessibility was computed using both the RNAfold and Sfold programs.

Figure S14: Boxplots showing the correlation between the mean accessibility and the mean % GC, and the mean number of SNPs and the mean % GC, calculated in non-overlapped windows of 5 nucleotides for both the functional and the broad set in the five major populations: African (AFR) using the Sfold (a) and RNAfold (b) programs, Ad Mixed American (AMR) using the Sfold (c) and RNAfold (d) programs, European (EUR) using the Sfold (e) and RNAfold (f) programs, East Asian (EAS) using the Sfold (g) and RNAfold (h) programs and South Asian populations (SAS) using the Sfold (i) and RNAfold (j) programs.

Figure S15: Differences between accessibility distributions of positions with or without SNPs for regions annoted as ESE (blue) and the rest of the regions (blue) for the functional and the broad sets. Each dot represents the probability of not having SNPs minus the probability of having SNPs within a given range of accessibilities (0-0.1, 0.1-0.2, 0.2-0.3, 0.3-0.4, 0.4-0.5, 0.5-0.6, 0.6-0.7, 0.7-0.8, 0.8-0.9, 0.9-1). Probabilities within ranges were calculated using the integrate.xy function on a density distribution (see material and methods). Vertical lines represent the confidence intervals estimated using a bootstrapping after 500 replicates. Accessibility was computed using both the Sfold (a) and Sfold (b) programs.

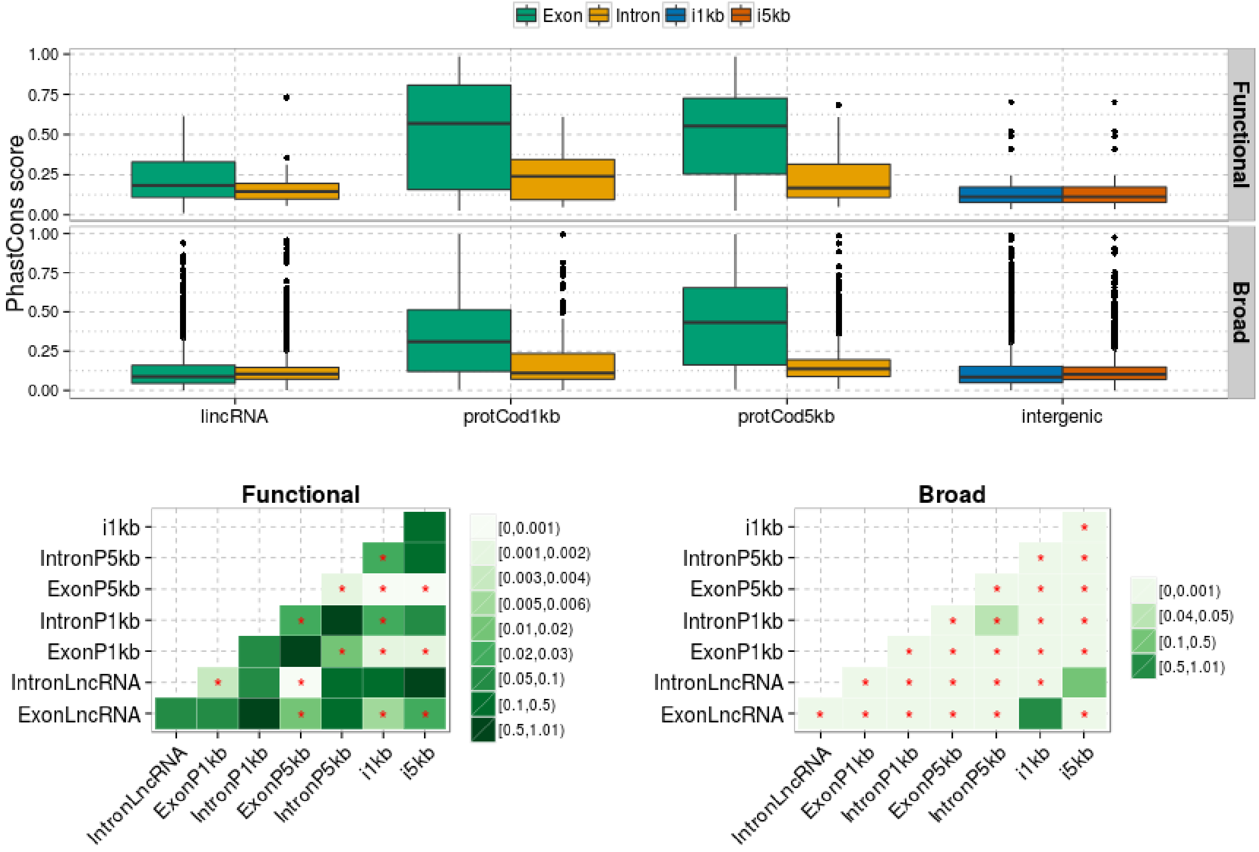

Sup. Fig. 1

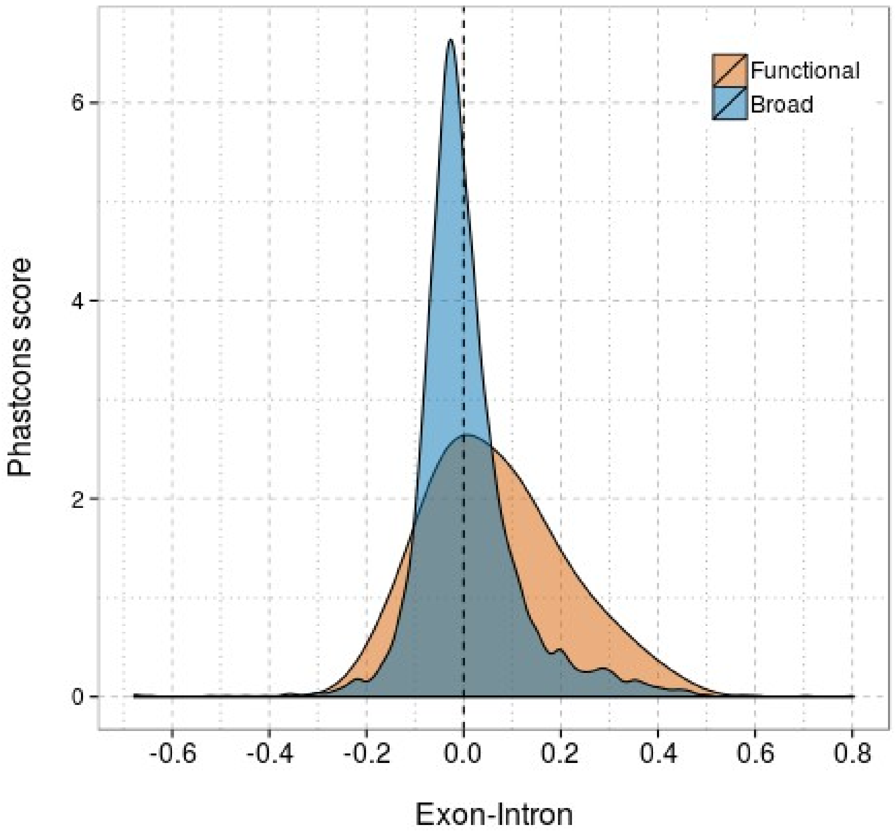

Sup. Fig. 2

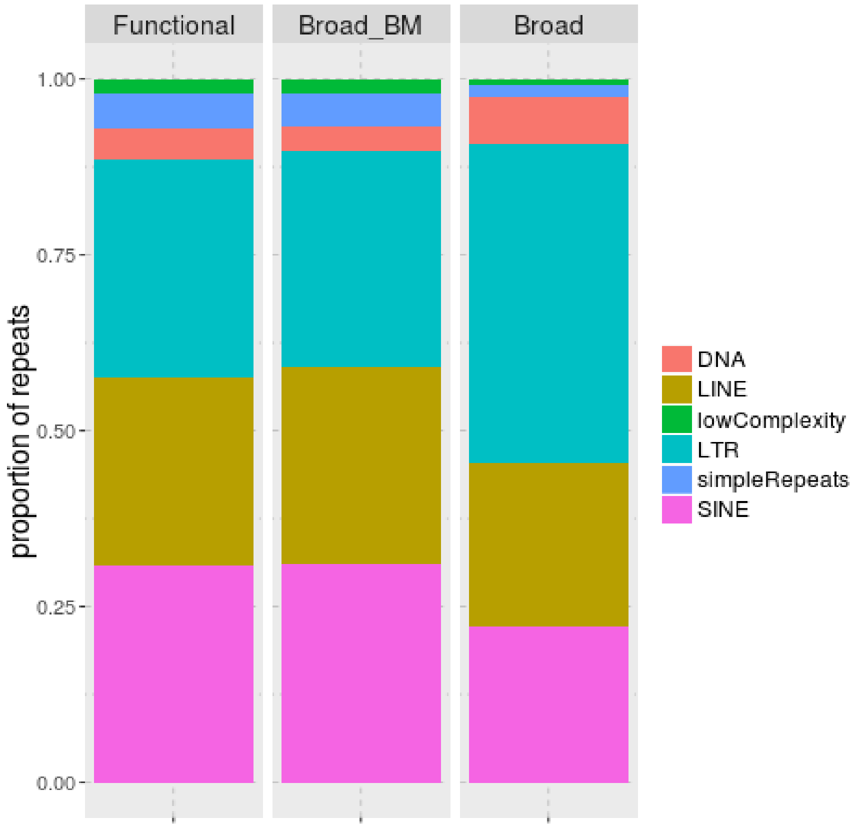

Sup. Fig. 3

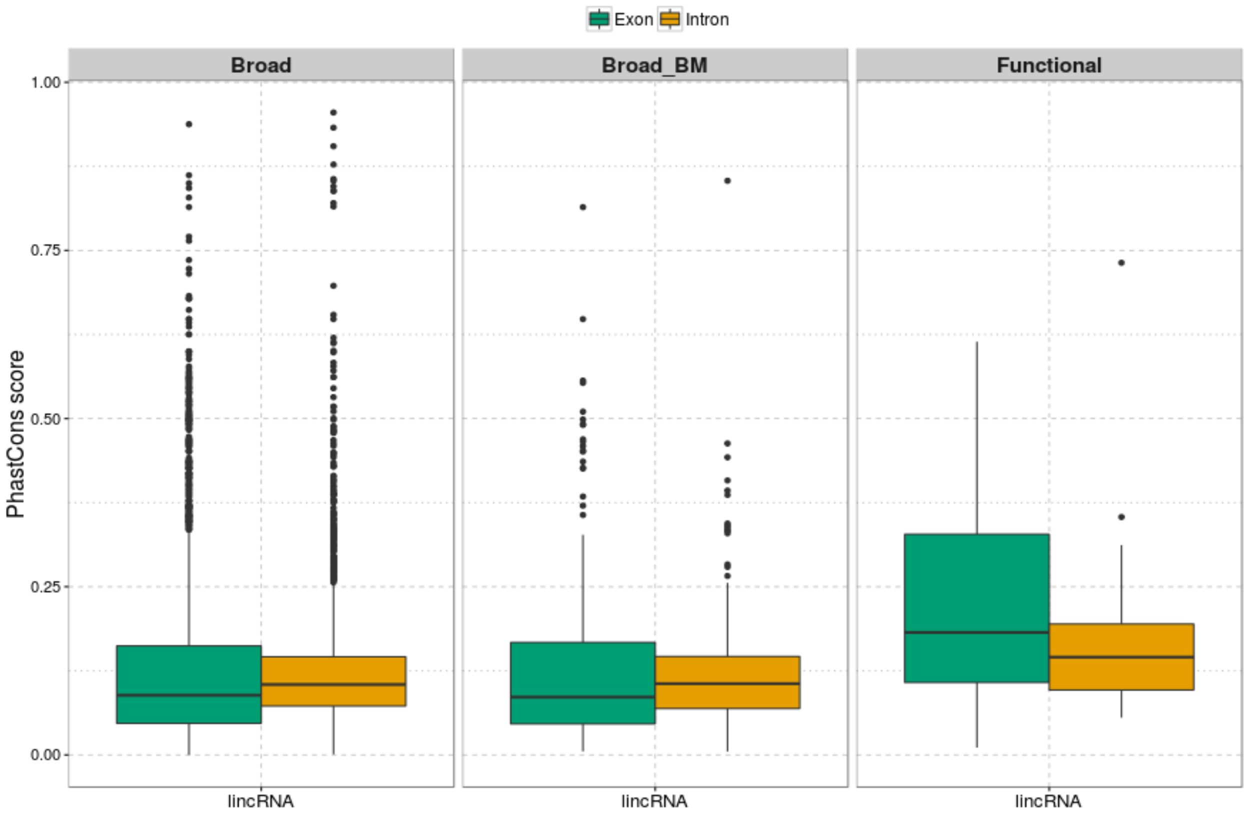

Sup. Fig. 4

Sup. Fig. 5

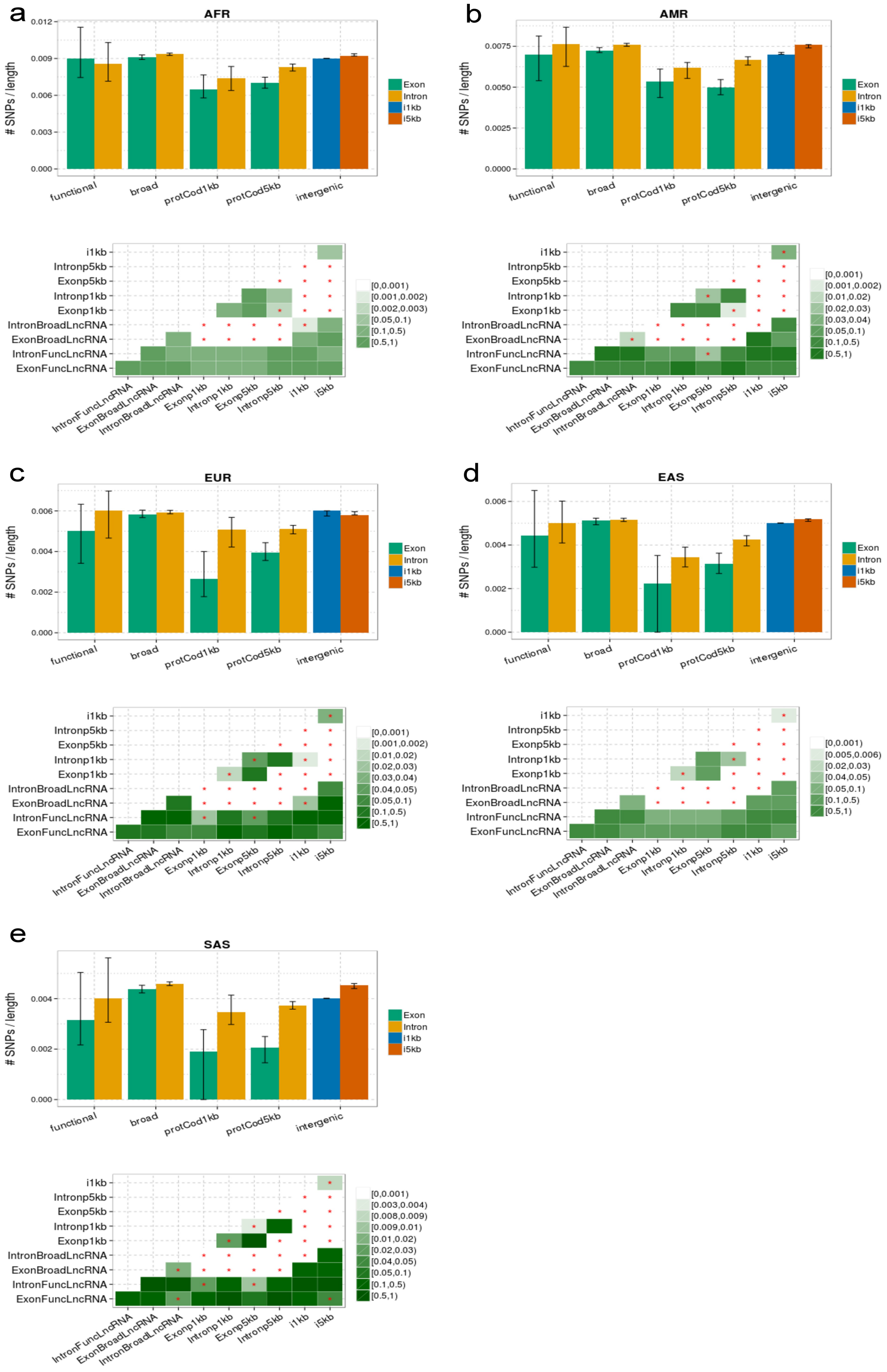
a
AFR
SNPs / length
functional
broad
protCod1kb
protCod5kb
intergenic
Exon
Intron
i1kb
i5kb
i1kb
Intronp5kb
Exonp5kb
Intronp1kb
Exonp1kb
IntronBroadLncRNA
ExonBroadLncRNA
IntronFuncLncRNA
ExonFuncLncRNA
b
AMR
c
EUR
d
EAS
e
SAS

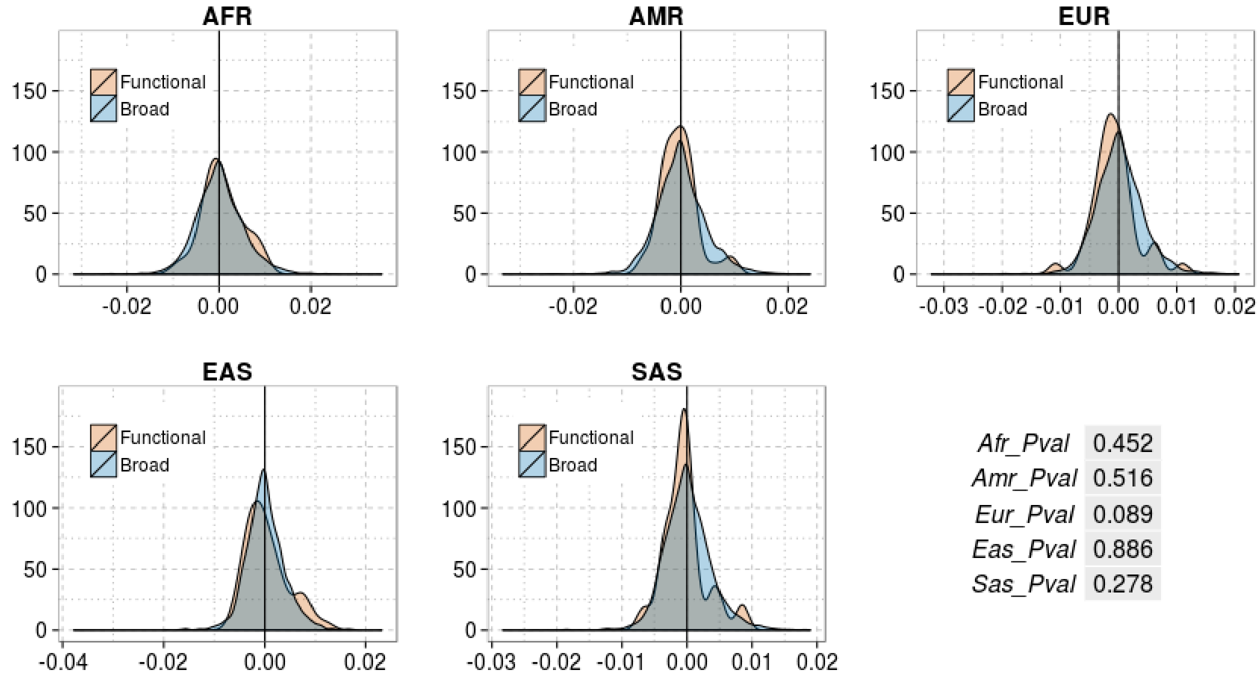
AFR
AMR
EUR
EAS
SAS
Functional
Broad
Afr_Pval 0.452
Amr_Pval 0.516
Eur_Pval 0.089
Eas_Pval 0.886
Sas_Pval 0.278

Sup. Fig. 6

a

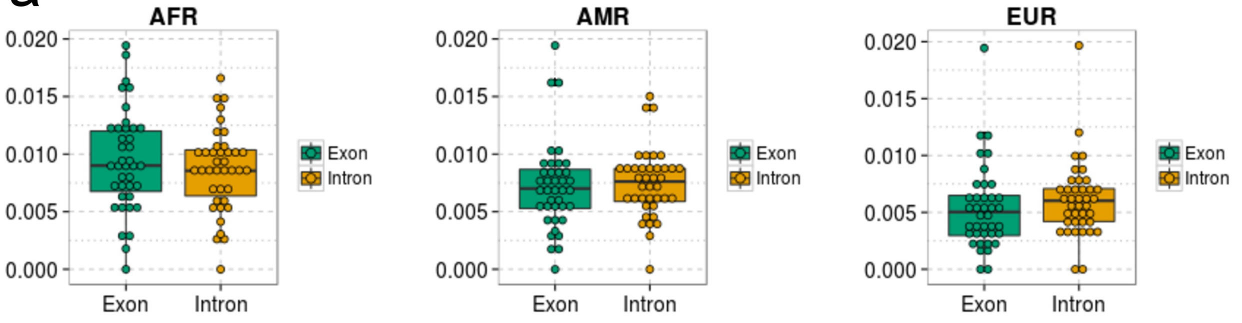

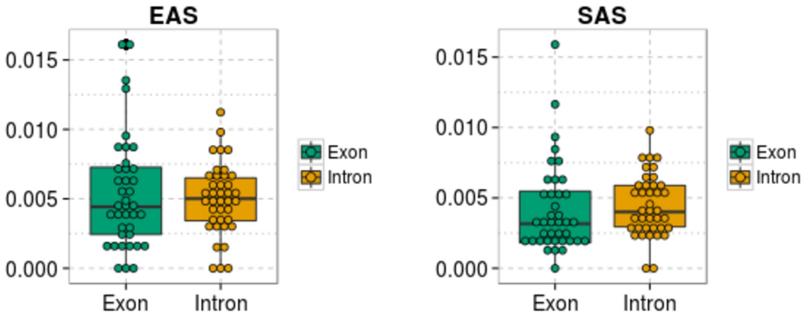

	P val	*Exons median*	*Introns median*
Afr	0.494	0.009	0.0086
Amr	0.427	0.007	0.0076
Eur	0.131	0.005	0.006
Eas	0.924	0.0044	0.005
Sas	0.11	0.0032	0.004

b

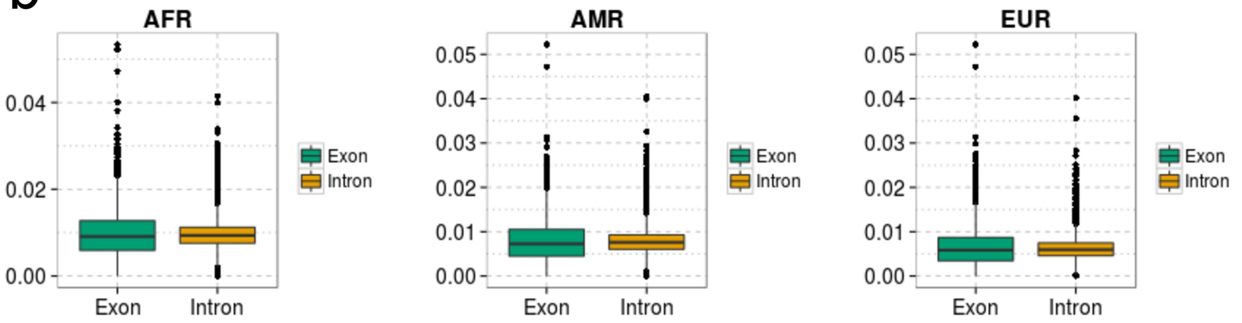

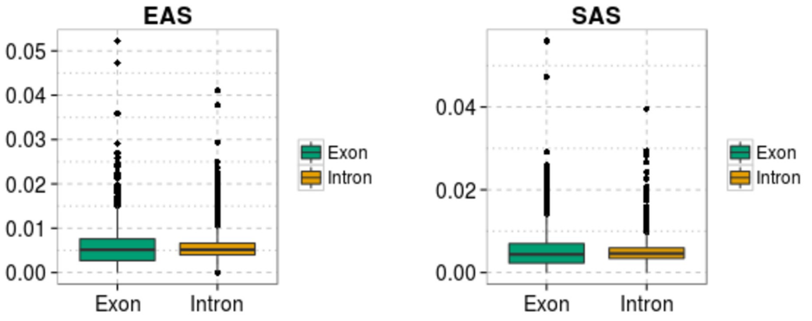

	P val	*Exons median*	*Introns median*
Afr	0.171	0.0091	0.0094
Amr	0.022	0.0073	0.0076
Eur	0.397	0.0058	0.0059
Eas	0.067	0.0051	0.0052
Sas	0.022	0.0044	0.0046

Sup. Fig. 7

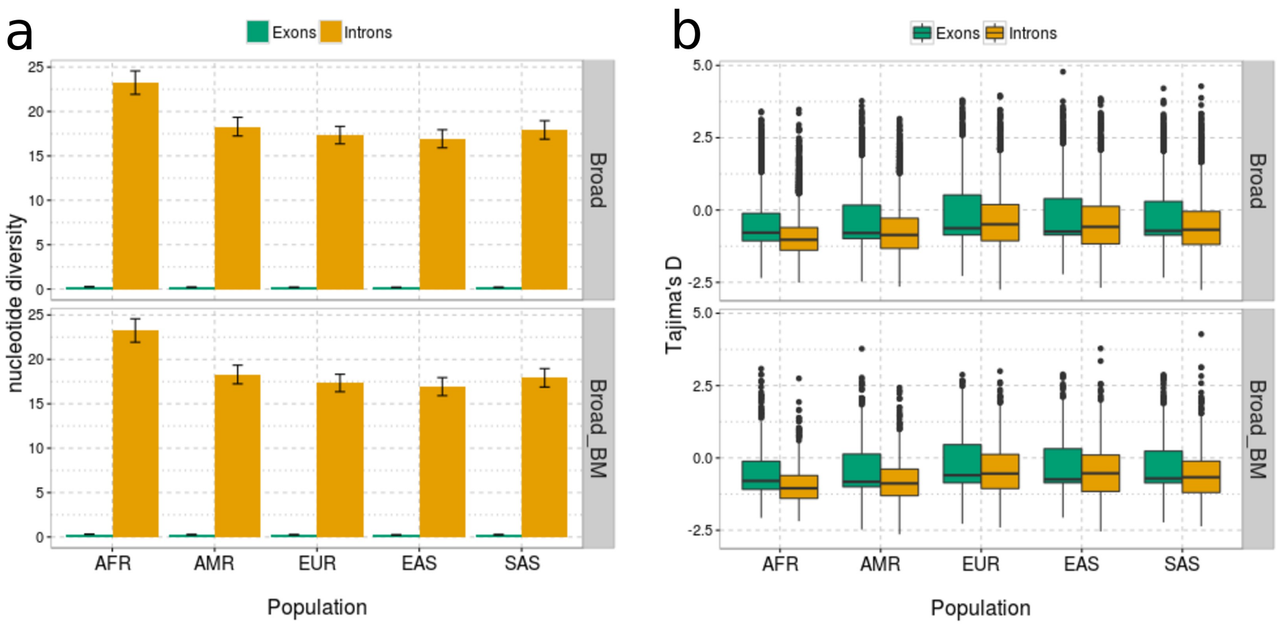

Sup. Fig. 8

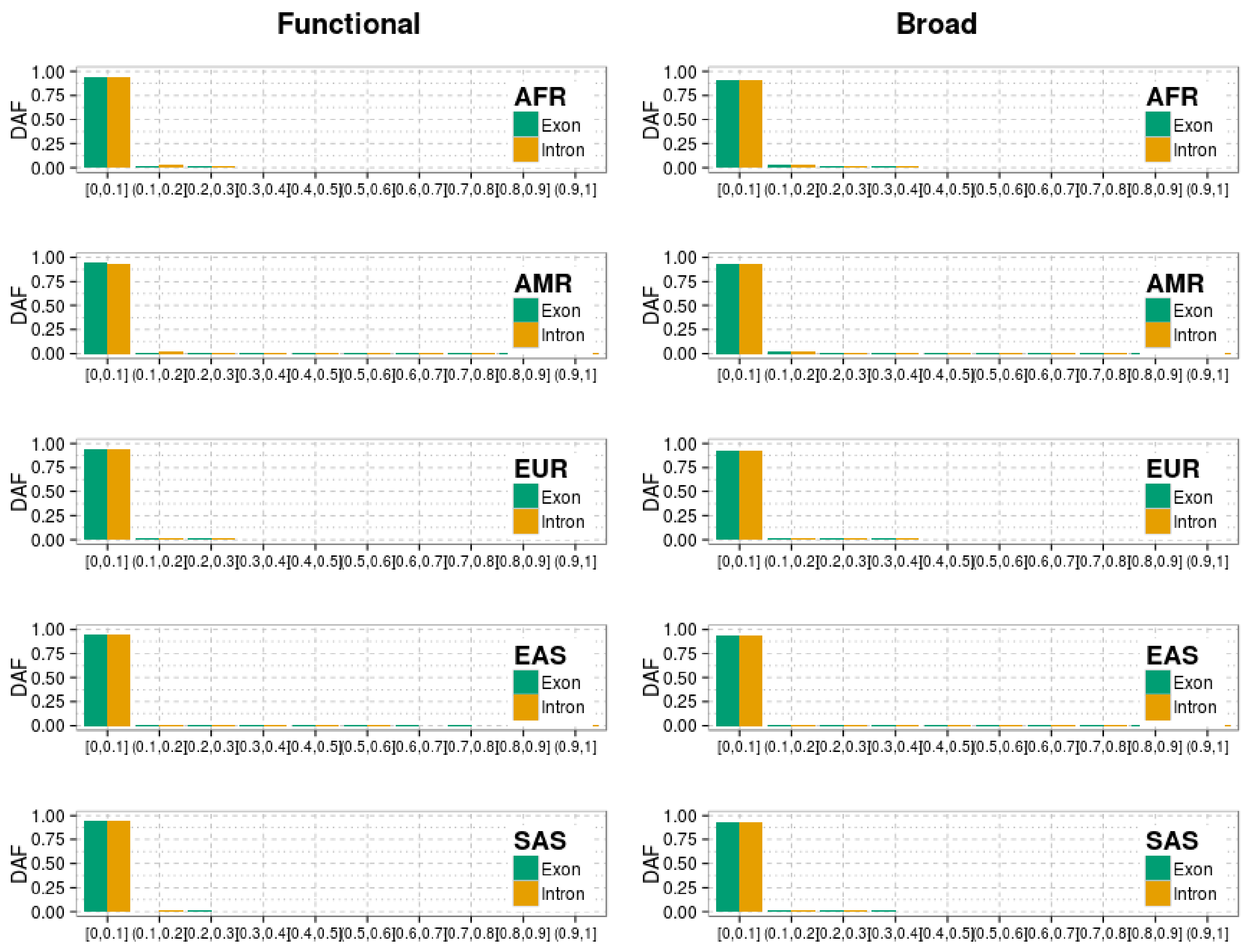

Sup. Fig. 9

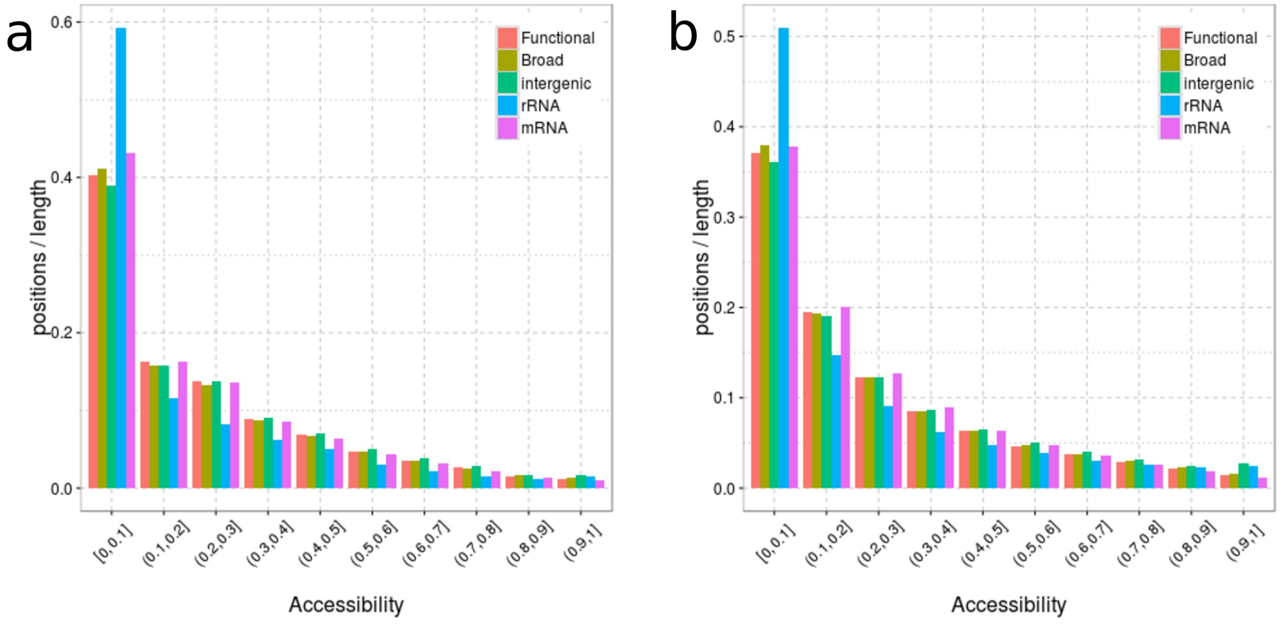

Sup. Fig. 10

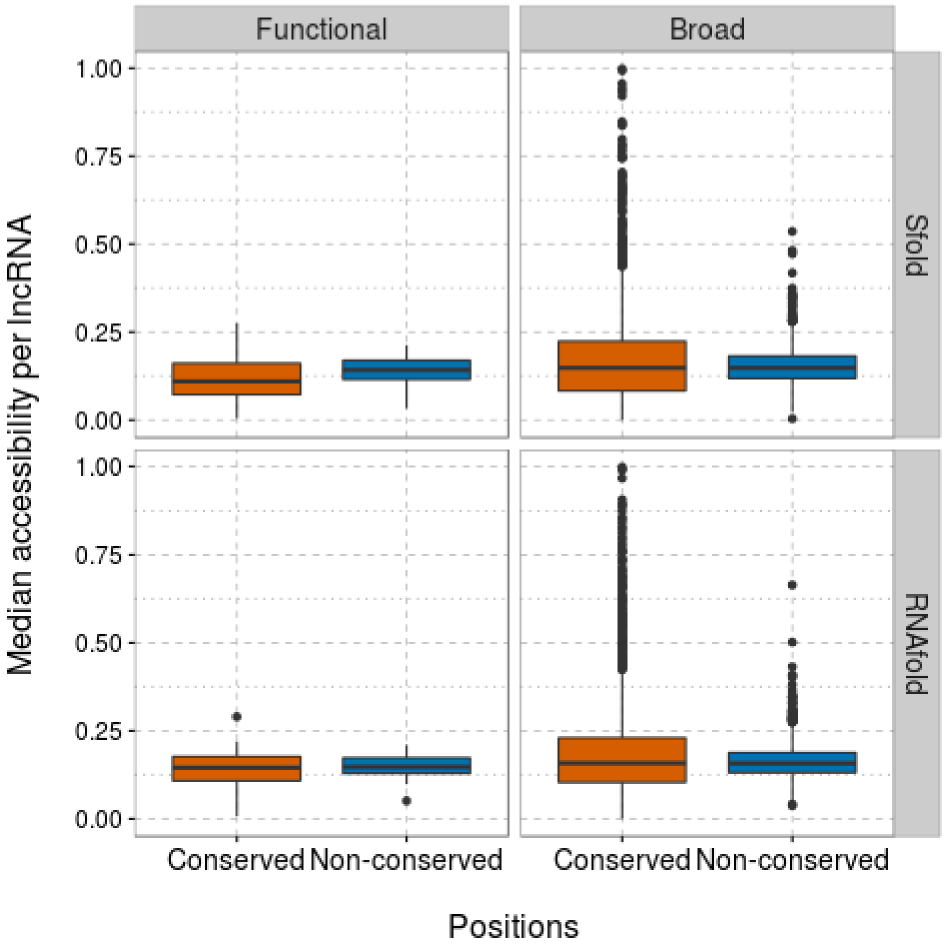

Sup. Fig. 11

Sup. Fig. 12

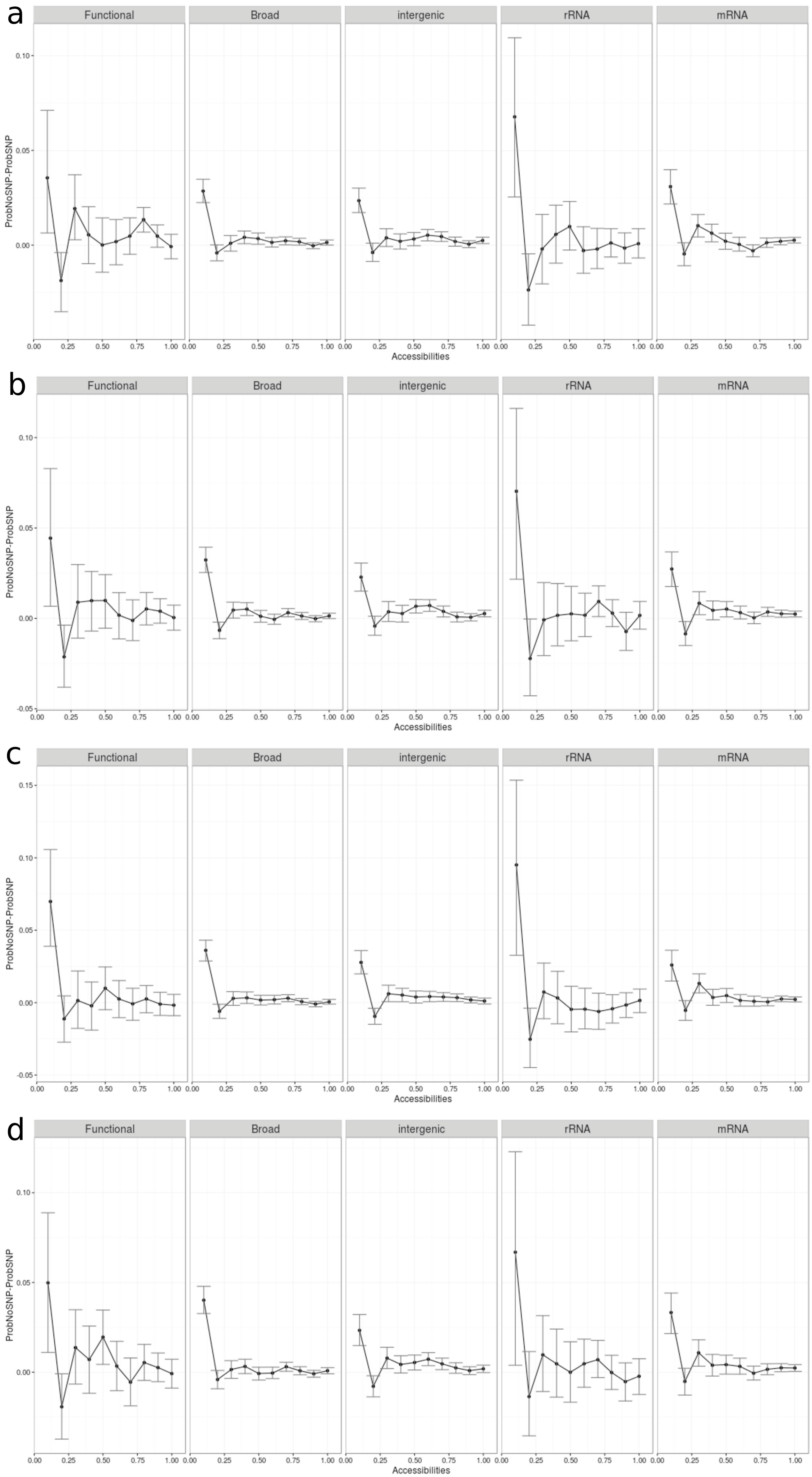

a
Functional
Broad
intergenic
rRNA
mRNA
ProbNoSNP-ProbSNP
Accessibilities
b
Functional
Broad
intergenic
rRNA
mRNA
ProbNoSNP-ProbSNP
Accessibilities
c
Functional
Broad
intergenic
rRNA
mRNA
ProbNoSNP-ProbSNP
Accessibilities
d
Functional
Broad
intergenic
rRNA
mRNA
ProbNoSNP-ProbSNP
Accessibilities

Sup. Fig. 12

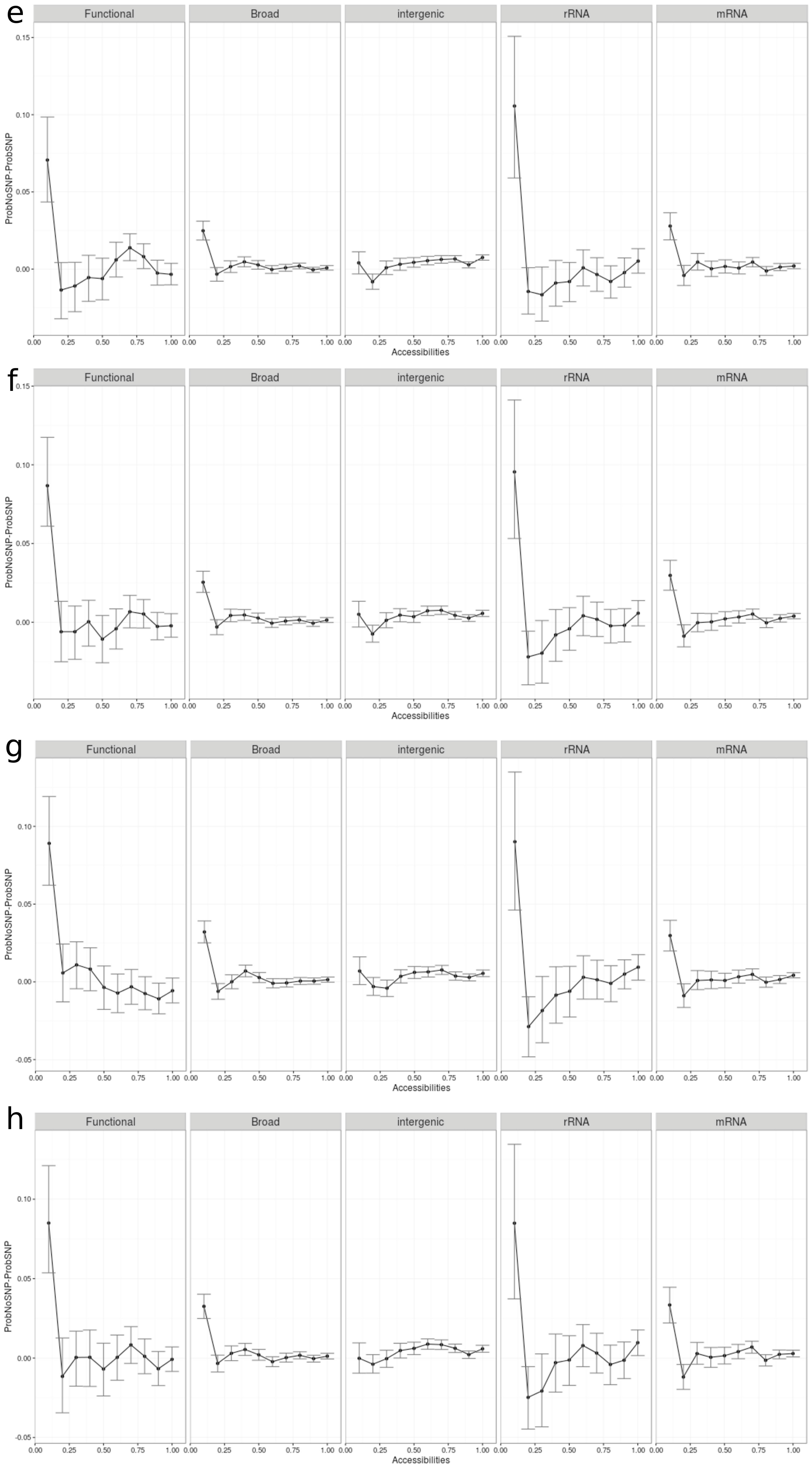
e
f
g
h
Functional
Broad
intergenic
rRNA
mRNA
ProbNoSNP-ProbSNP
Accessibilities
0.15
0.10
0.05
0.00
-0.05
0.00
0.25
0.50
0.75
1.00

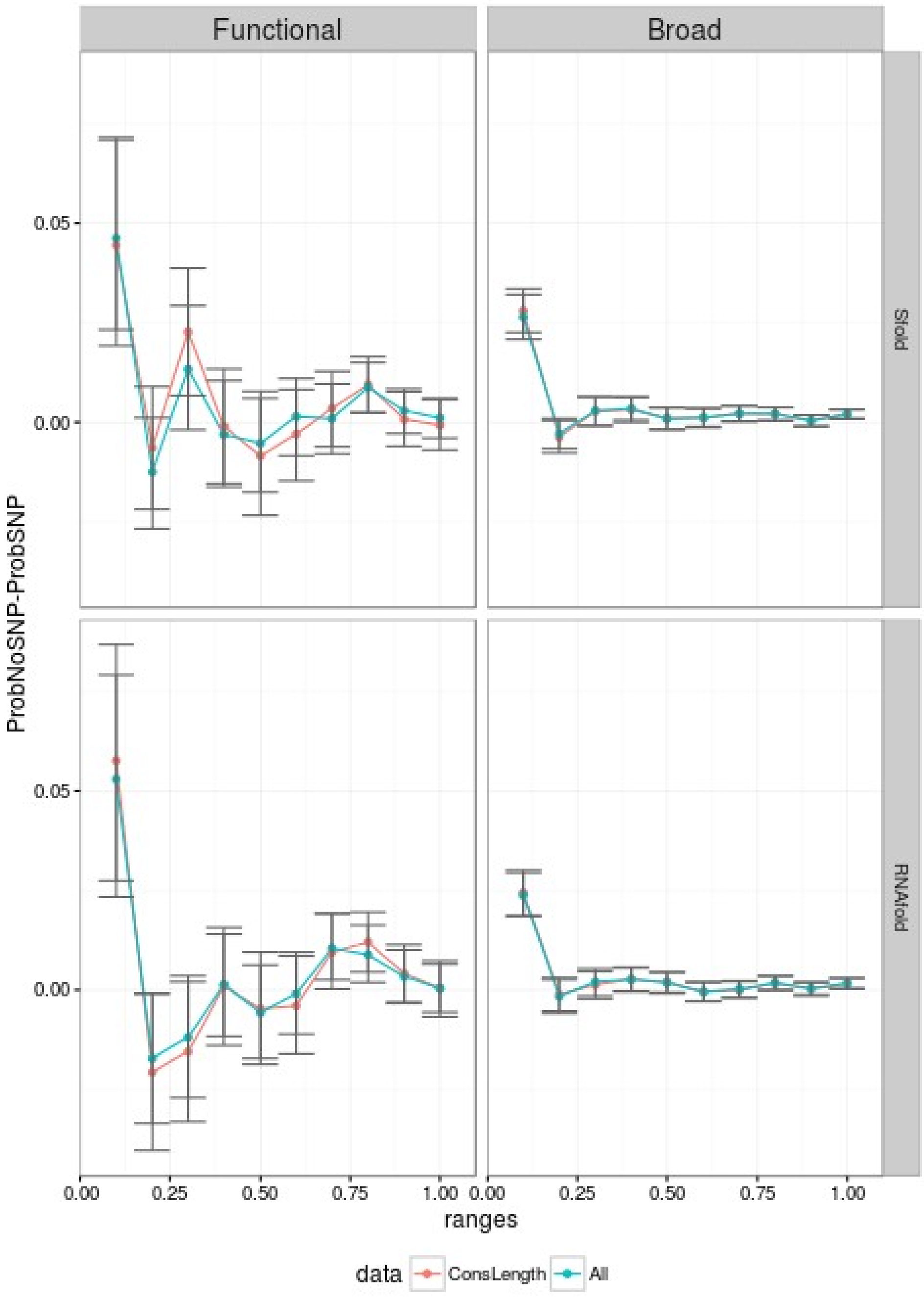

Functional
Broad
Stoid
RNAfold
ProbNoSNP-ProbSNP
0.05
0.00
0.05
0.00
0.00
0.25
0.50
0.75
1.00
0.00
0.25
0.50
0.75
1.00
ranges
data
ConsLength
All

Sup. Fig. 13

Sup. Fig. 14

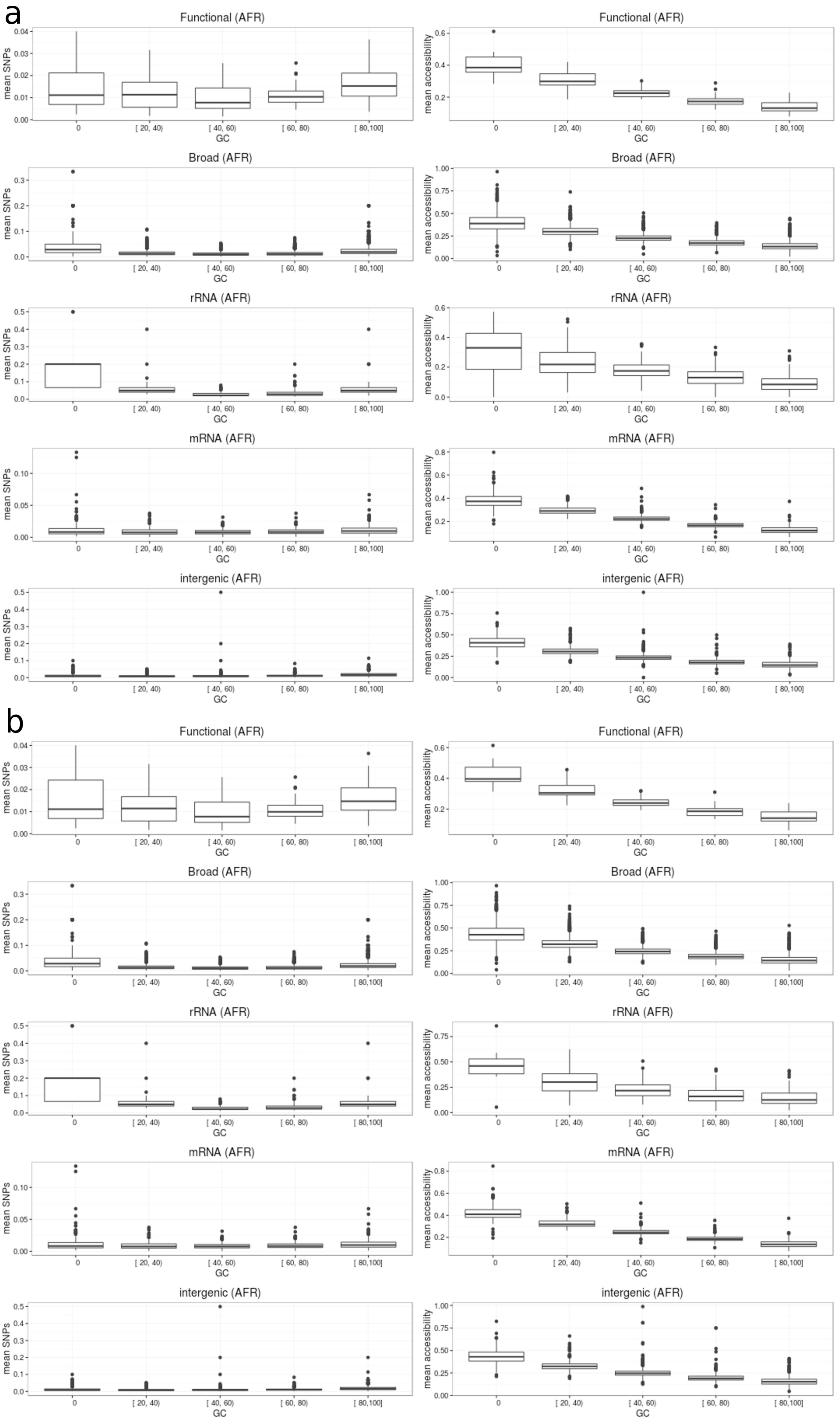
a
Functional (AFR)
mean SNPs
mean accessibility
GC
0
[20, 40)
[40, 60)
[60, 80)
[80,100]
Broad (AFR)
rRNA (AFR)
mRNA (AFR)
intergenic (AFR)
b
Functional (AFR)
Broad (AFR)
rRNA (AFR)
mRNA (AFR)
intergenic (AFR)

Sup. Fig. 14

c

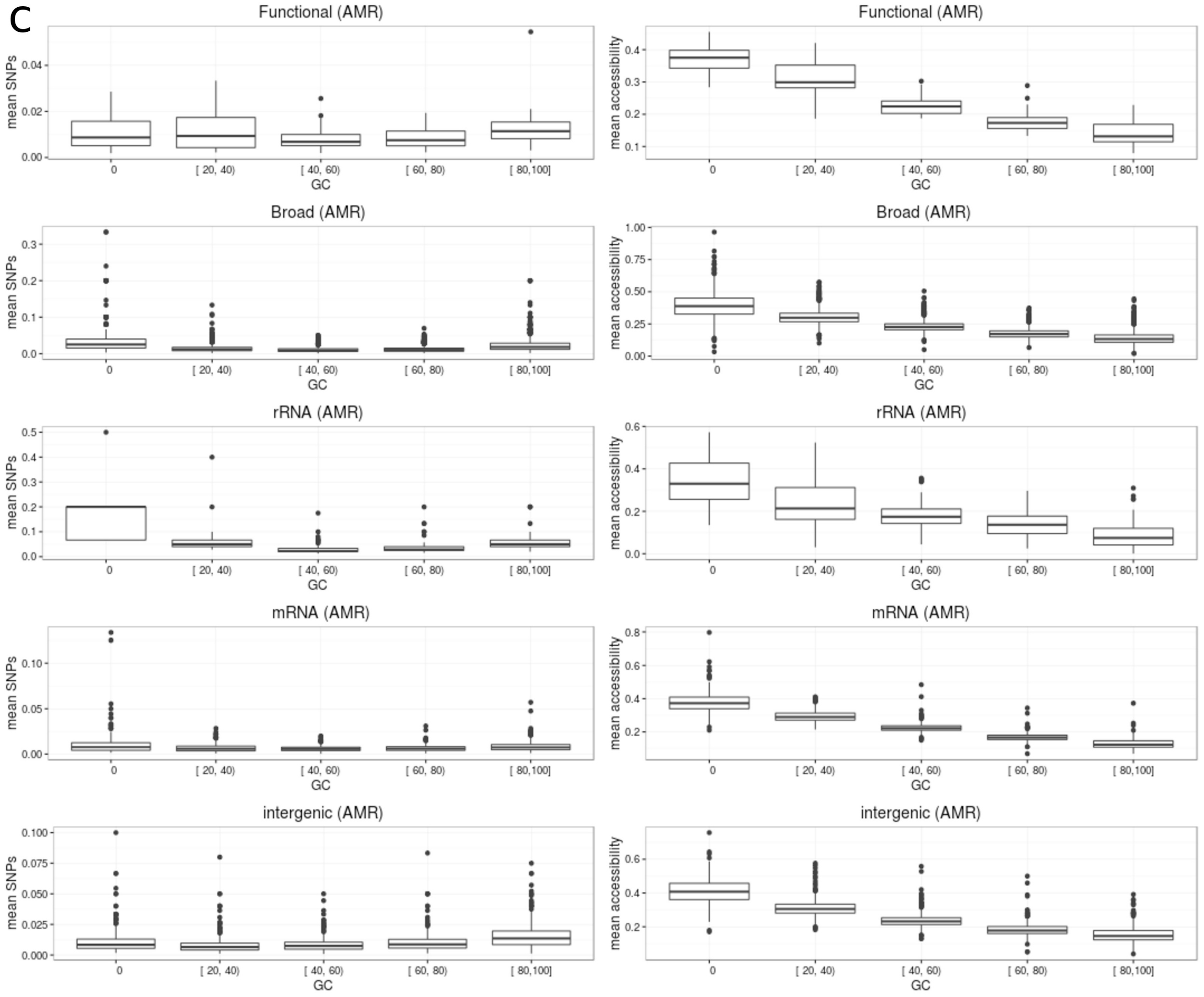
Functional (AMR)
Broad (AMR)
rRNA (AMR)
mRNA (AMR)
intergenic (AMR)
mean SNPs
mean accessibility
GC
0
[20, 40)
[40, 60)
[60, 80)
[80,100]

d

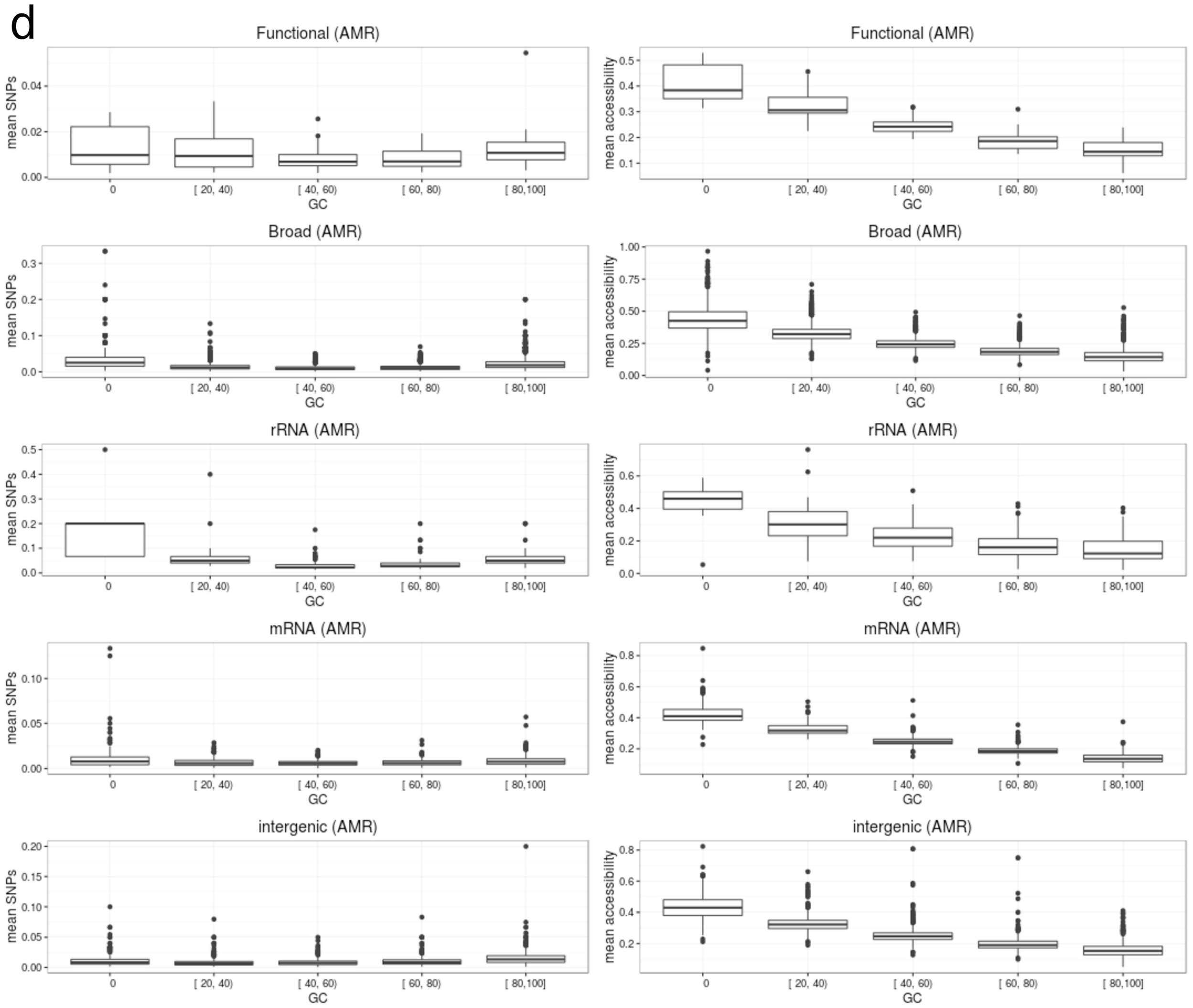
Functional (AMR)
Broad (AMR)
rRNA (AMR)
mRNA (AMR)
intergenic (AMR)
mean SNPs
mean accessibility
GC
0
[20, 40)
[40, 60)
[60, 80)
[80,100]

Sup. Fig.14

e

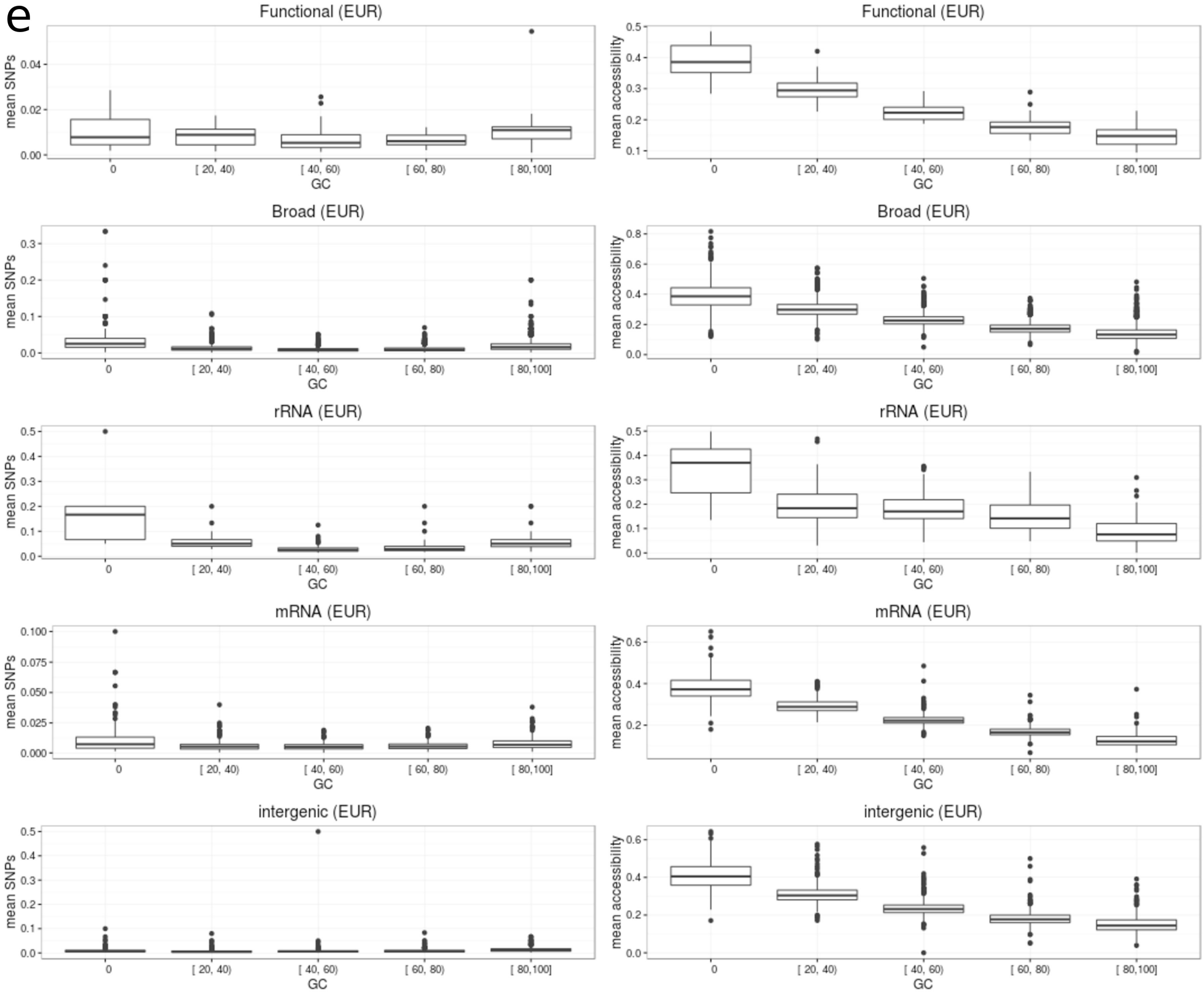
Functional (EUR)
mean SNPs
mean accessibility
Broad (EUR)
rRNA (EUR)
mRNA (EUR)
intergenic (EUR)
0
[20, 40)
[40, 60)
[60, 80)
[80,100]
GC

f

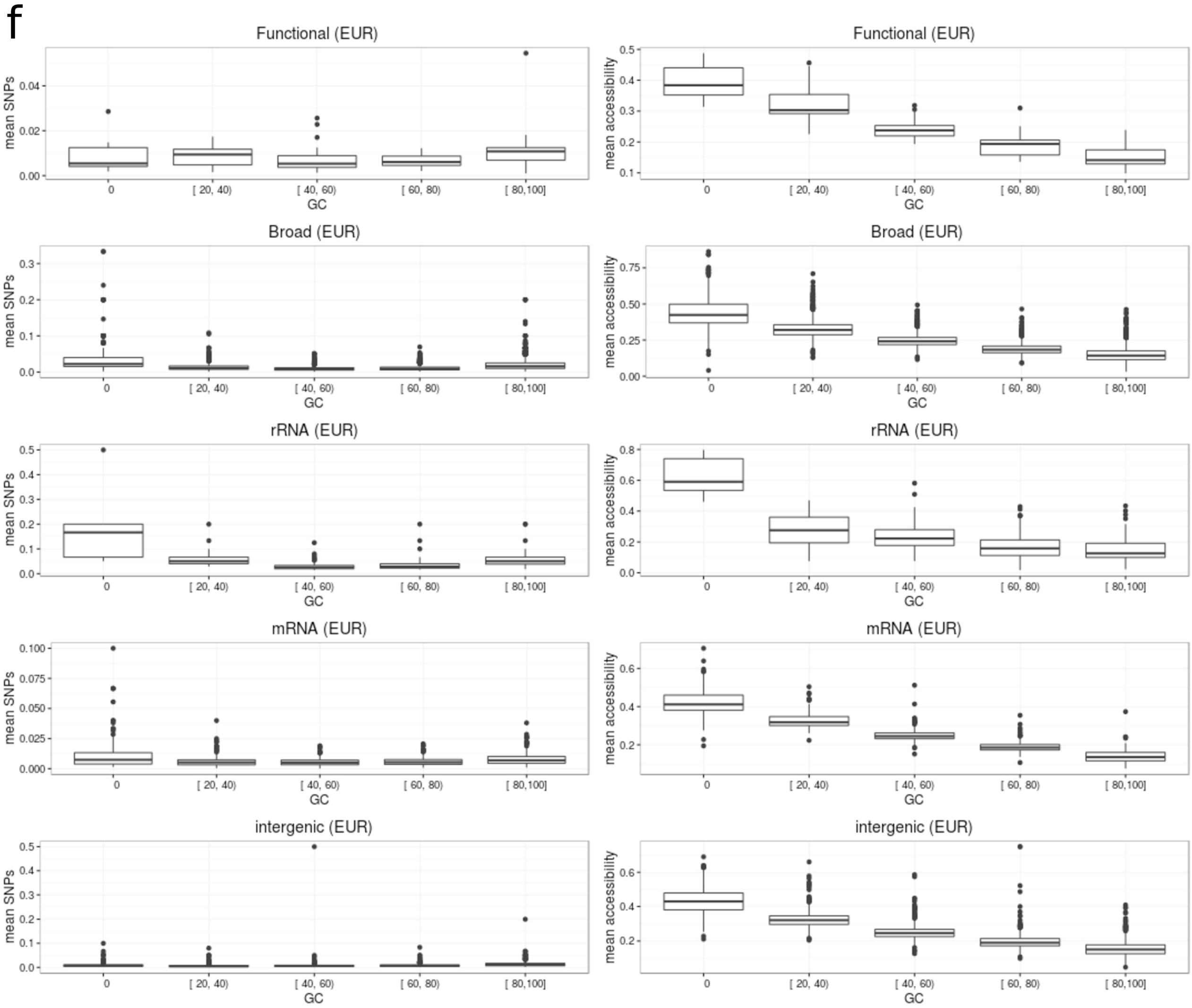
Functional (EUR)
mean SNPs
mean accessibility
Broad (EUR)
rRNA (EUR)
mRNA (EUR)
intergenic (EUR)
0
[20, 40)
[40, 60)
[60, 80)
[80,100]
GC

Sup. Fig. 14

i

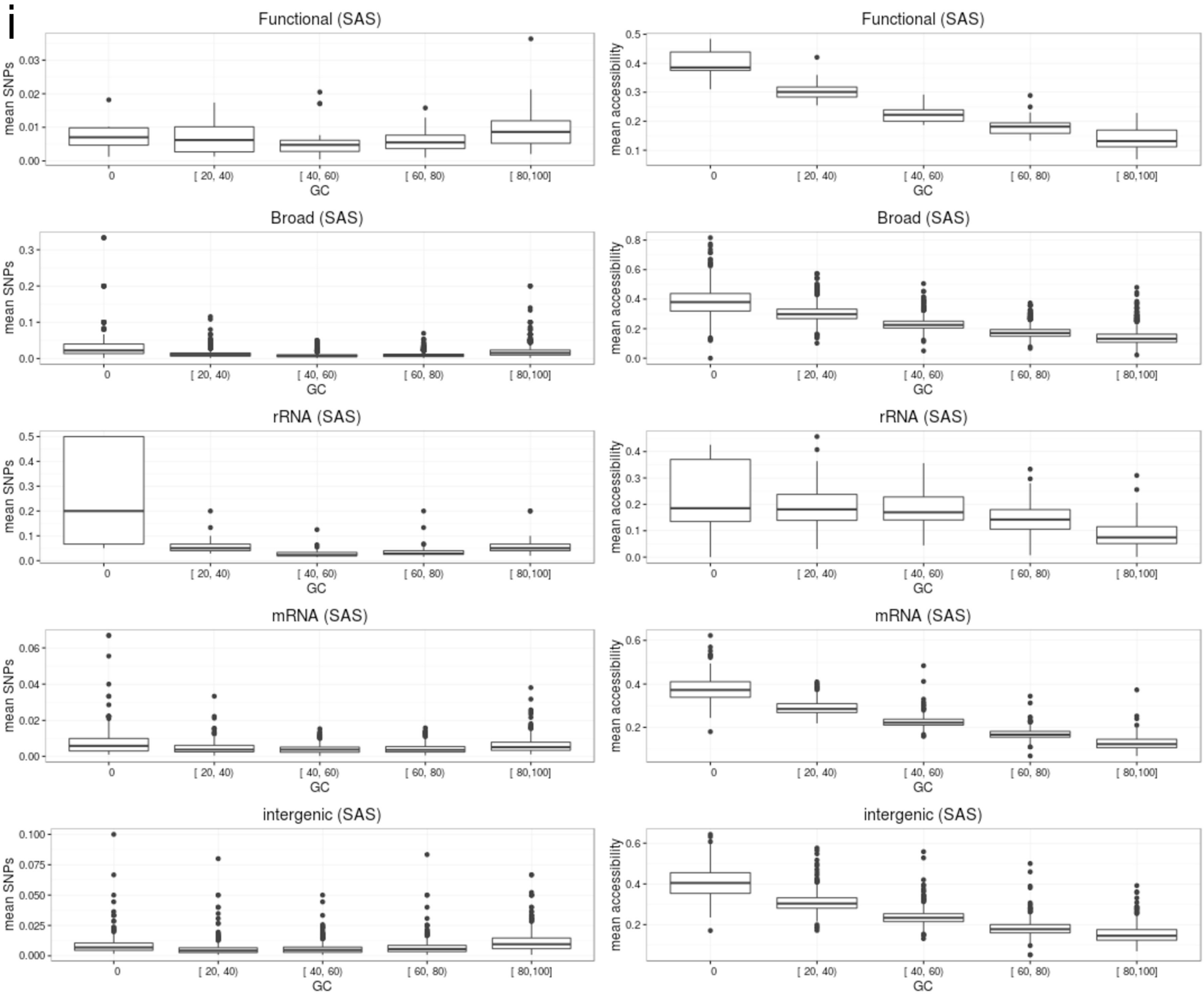

Functional (SAS)
Broad (SAS)
rRNA (SAS)
mRNA (SAS)
intergenic (SAS)
mean SNPs
mean accessibility
GC
0
[20, 40)
[40, 60)
[60, 80)
[80,100]

j

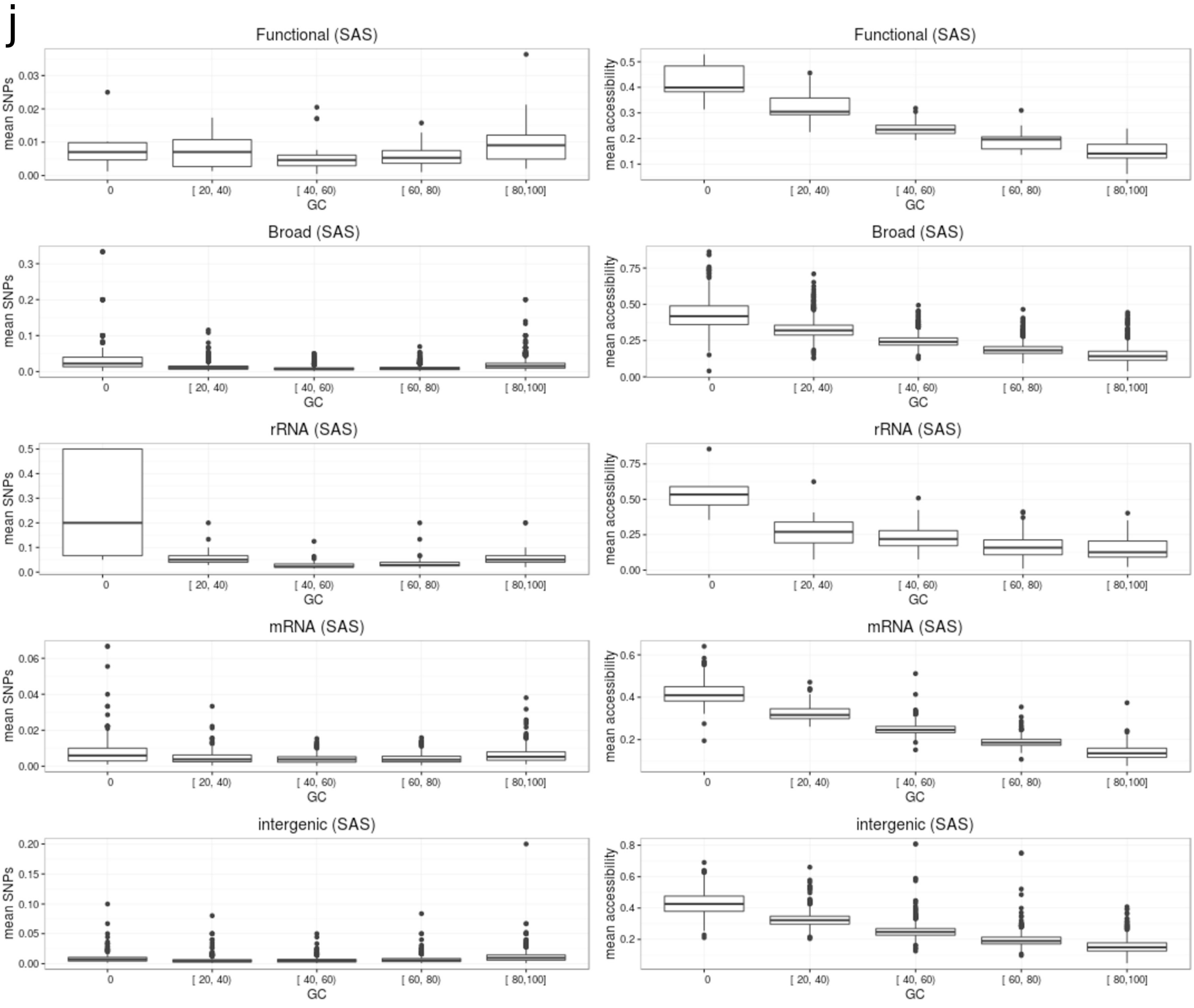

Functional (SAS)
Broad (SAS)
rRNA (SAS)
mRNA (SAS)
intergenic (SAS)
mean SNPs
mean accessibility
GC
0
[20, 40)
[40, 60)
[60, 80)
[80,100]

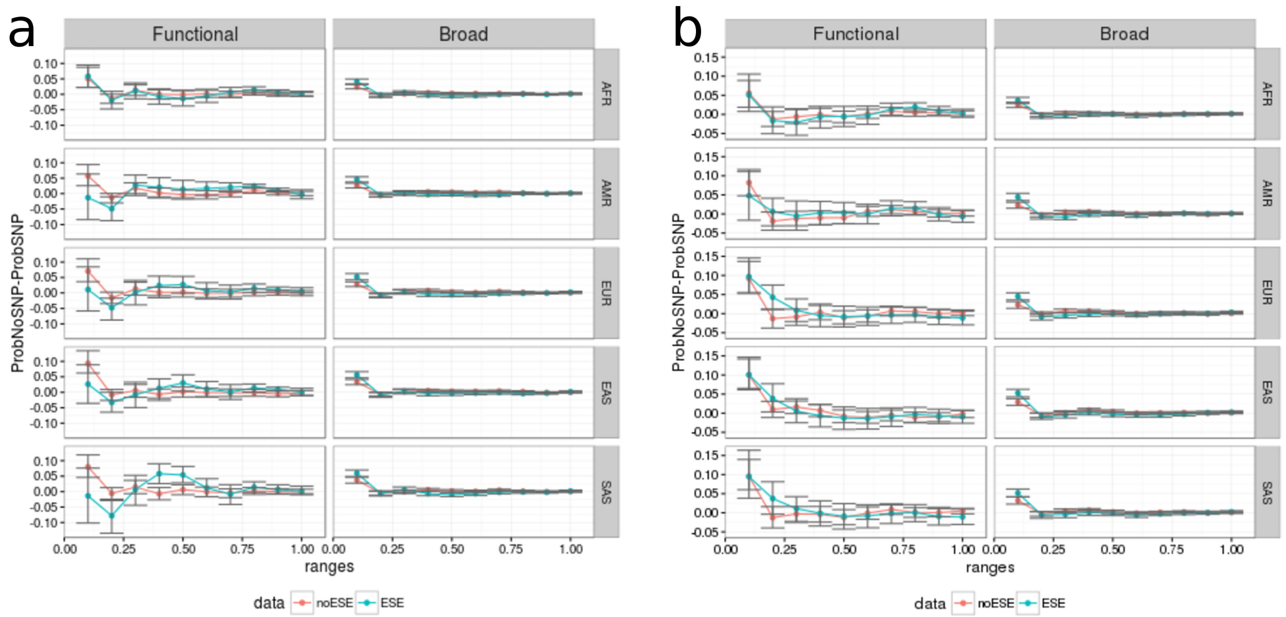
a
b
Functional
Broad
AFR
AMR
EUR
EAS
SAS
ProbNoSNP-ProbSNP
ranges
data
noESE
ESE

Sup. Fig. 15
